# Integrated Source Case Investigation for Tuberculosis (TB) and HIV in the Caregivers and Household Contacts of Hospitalised Young Children Diagnosed with TB in South Africa: An Observational Study

**DOI:** 10.1371/journal.pone.0137518

**Published:** 2015-09-17

**Authors:** Sanjay G. Lala, Kristen M. Little, Nkeko Tshabangu, David P. Moore, Reginah Msandiwa, Martin van der Watt, Richard E. Chaisson, Neil A. Martinson

**Affiliations:** 1 Department of Paediatrics and Child Health, Chris Hani Baragwanath Academic Hospital and University of the Witwatersrand, Johannesburg, South Africa; 2 Department of Epidemiology, Johns Hopkins Bloomberg School of Public Health, Baltimore, Maryland, United States of America; 3 Perinatal HIV Research Unit, University of the Witwatersrand, Johannesburg, South Africa; 4 Center for TB Research, Johns Hopkins University School of Medicine, Baltimore, Maryland, United States of America; 5 Department of Science and Technology/National Research Foundation (DST/NRF) Centre of Excellence for Biomedical TB Research, University of the Witwatersrand, Johannesburg, South Africa; University of Cape Town, SOUTH AFRICA

## Abstract

**Background:**

Contact tracing, to identify source cases with untreated tuberculosis (TB), is rarely performed in high disease burden settings when the index case is a young child with TB. As TB is strongly associated with HIV infection in these settings, we used source case investigation to determine the prevalence of undiagnosed TB and HIV in the caregivers and household contacts of hospitalised young children diagnosed with TB in South Africa.

**Methods:**

Caregivers and household contacts of 576 young children (age ≤7 years) with TB diagnosed between May 2010 and August 2012 were screened for TB and HIV. The primary outcome was the detection of laboratory-confirmed, newly-diagnosed TB disease and/or HIV-infection in close contacts.

**Results:**

Of 576 caregivers, 301 (52·3%) self-reported HIV-positivity. Newly-diagnosed HIV infection was detected in 63 (22·9%) of the remaining 275 caregivers who self-reported an unknown or negative HIV status. Screening identified 133 (23·1%) caregivers eligible for immediate anti-retroviral therapy (ART). Newly-diagnosed TB disease was detected in 23 (4·0%) caregivers. In non-caregiver household contacts (n = 1341), the prevalence of newly-diagnosed HIV infection and TB disease was 10·0% and 3·2% respectively. On average, screening contacts of every nine children with TB resulted in the identification of one case of newly-diagnosed TB disease, three cases of newly diagnosed HIV-infection, and three HIV-infected persons eligible for ART.

**Conclusion:**

In high burden countries, source case investigation yields high rates of previously undiagnosed HIV and TB infection in the close contacts of hospitalised young children diagnosed with TB. Furthermore, integrated screening identifies many individuals who are eligible for immediate ART. Similar studies, with costing analyses, should be undertaken in other high burden settings–integrated source case investigation for TB and HIV should be routinely undertaken if our findings are confirmed.

## Introduction

To combat the dual epidemics of tuberculosis (TB) and HIV, the World Health Organisation (WHO) recommends a series of integrated tasks including intensified case-finding for TB, the provision of antiretroviral therapy (ART) to HIV-infected individuals, and the use of preventive TB treatment [1]. Intensified case-finding or contact tracing aims to identify undiagnosed TB disease, or latent infection, in contacts recently exposed to an infectious TB case so that these contacts can benefit from treatment or preventive therapy. Contact tracing is considered to be essential for controlling the TB epidemic and is prioritised as part of TB control efforts in high-income countries, although it is not routinely performed in high burden, resourced-limited settings [2, 3].

Typically, contact tracing is initiated after TB is confirmed in an index case—usually an adult with infectious TB. However, when the index case is a young child with suspected (rather than confirmed) tuberculosis, contact tracing (variously known as source case investigation, reverse contact tracing or ascending surveys [4–8]) is reported on less frequently. The reluctance to undertake source case investigation is probably based on the assumption that TB is diagnosed in children after the disease is confirmed in an adult household suspect [9]. The difficulty of confirming TB disease in children—only 20–30% of children with TB have laboratory confirmation [10, 11]–is another important reason that raises concerns about the effectiveness and costs of source case investigation.

Although it is widely accepted that young children usually acquire TB infection from an infectious adult living in the household [12, 13], a household TB source is reported in only 34-50% of culture confirmed childhood TB cases in high burden settings [14, 15]. In addition, a spatial and temporal transmission analysis performed in a South African township shows that TB is often diagnosed in children before adults who live on the same residential plot [16]. There may be several reasons for this but one possibility is that TB disease remains undiagnosed in household adult contacts. Household contacts may avoid visiting health care facilities because of the stigma associated with TB diagnosis and/or the fear that they may be co-infected with HIV [17, 18]; alternatively, health care providers may fail to diagnose TB disease in household suspects [19].

In South Africa, TB is diagnosed often in acutely-ill, hospitalised young children [14, 15, 20]. In this situation, source case investigation could be effectively initiated because caregivers can be opportunistically screened for TB and HIV when they accompany their children to hospital. We therefore conducted a prospective source case investigation (SCI) study, using an integrated, replicable and well defined HIV and TB screening programme (which included a determination for ART in HIV-infected persons), to systematically screen both symptomatic and asymptomatic caregivers and household contacts of hospitalised young children with TB.

## Materials and Methods

### Participants

We recruited the primary caregivers and household contacts of children ≤7 years who were diagnosed with TB during an admission for an acute illness to the Chris Hani Baragwanath Academic Hospital (CHBAH) in Soweto, South Africa during May 2010 to August 2012. We included children ≤7 years because we assumed that most TB exposure probably occurs in the household in children ≤7 years because South African children typically start formal schooling in the calendar year that they are 7-years-old. We were interested to ascertain whether children with TB could be used as sentinel cases to identify household TB transmitters before infecting others.

From notification data, 1261 hospitalised children aged ≤7 years were diagnosed with TB during the study period. Participants were recruited if our study nurses were able to contact the child’s caregiver during office hours, and we recruited 576 children in total. We did not recruit participants after-hours or over weekends, and some children could have been admitted and discharged over the weekend. Some participants could have been from outside the study area, and some could have refused or not had a caregiver available. Unfortunately we did not collect data on the children and their caregivers who were not eligible or not recruited.

The diagnosis of TB was made by the attending paediatrician [S1 File]. We defined children whose gastric aspirates were positive for acid-fast bacilli (AFB) on smear microscopy, and/or positive for *Mycobacterium tuberculosis* culture, or who had histological features typical of TB (e.g. lymph node biopsy), as having laboratory-confirmed TB. Our study did not conduct TB testing for sentinel cases, but retrieved results of patients who had been investigated routinely in the hospital. At CHBAH, at least two (but usually three) gastric aspirates are collected from children. Although we do not report the data in our manuscript, most children had gastric aspirates taken for microscopy and culture. A child who was notified for TB and initiated on TB treatment was considered to be an index (sentinel) case of childhood TB. The primary caregiver was defined as the person who brought the child to hospital initially, and household contacts were defined as any individual who slept in the same residence at least 2 nights per week. Caregivers who lived more than 80 km from the hospital or who did not live with other household members were not enrolled. We included any household contact who consented to participation. For each child included in the study, we did at least three recruitment visits to recruit household contacts, attempting to do these visits at different times of the day with at least one of the recruitment visits being on the weekend.

### Study procedures

All study procedures, including data abstraction from clinical records, administration of structured interviews, and counselling and testing for HIV and TB, were performed after obtaining written informed consent from the caregivers and household contacts. In households, written assent was obtained from children between 7–14 years of age.

We obtained informed consent from the next of kin, caretakers, or guardians on behalf of the minors/children enrolled in our study. In virtually all instances, consent was obtained from the mothers of the children. The consent obtained on behalf of the children enrolled was written. No verbal consents were obtained. All consents were recorded on an approved parental informed consent document. If someone did not agree to participate in the study, we collected no information about them. The ethics committees of the University of the Witwatersrand and Johns Hopkins School of Medicine, and the Research Committee of CHBAH, approved this study.

#### Children with sentinel TB

Data was abstracted from the clinical record and laboratory results were obtained from electronic records. We interviewed the caregiver to record the child’s symptoms and possible TB exposure in the household [S2 File].

#### Caregivers and household contacts of children with sentinel TB

A structured interview [S3 and S4 Files] focusing on TB symptoms and duration (cough, coughing sputum or blood, weight loss, fever, soaking night sweats, shortness of breath and loss of appetite), demographics, smoking history, employment, and income was administered to contacts. Caregivers were interviewed at the hospital and household contacts at the household. Information describing the number of rooms, windows and doors in each household dwelling was collected [S5 File].

#### TB and HIV testing

A single spot sputum sample was obtained for smear microscopy and submitted for auramine staining and liquid culture using the Mycobacteria Growth Indicator Tube (MGIT) System (BD, Franklin Lakes, NJ, USA). Genotypic resistance to isoniazid and rifampicin was detected using the GenoType MTBDRplus test (Hain Lifescience GmbH, Germany). Although the WHO recommends that two sputa samples (including an early morning or overnight sputum sample) be collected, the logistics of performing such a large household study (that would have required additional household visits) precluded such collections. HIV testing was performed using rapid HIV tests (FirstResponse [PMC Medical, India] and Sensa [Pantech, Durban, South Africa]) or salivary HIV enzyme immunoassay (OraSure, Bethlehem, PA); initially positive HIV results were confirmed by ELISA. Participants with self-reported or newly diagnosed HIV-infection had CD4 counts performed. The only tests that were done in the field were rapid HIV tests on participants who consented for this. Specimens for all other tests were collected in the field but tests performed in the laboratory. Those contacts with positive TB results and/or those HIV-infected contacts eligible for ART initiation (according to the South African guidelines [21]) were referred to clinics for treatment and notification.

Heel prick HIV DNA PCR was offered to children aged 6–18 months. Household children ≤6 months were referred to the CHBAH for clinical examination, blood draw and collection of induced sputum. Children aged 6 months to 5 years who resided in households with a sputum positive (smear or culture) member were referred to clinics for isoniazid prophylaxis or TB treatment.

For caregivers and household contacts, *newly-diagnosed TB disease* was defined by the presence of laboratory-confirmed TB in an individual who did not self-report TB and was not receiving concurrent TB treatment. *Laboratory-confirmed TB* was defined as a positive sputum microscopy and/or *M*. *tuberculosis* culture result, or AFB or *M*. *tuberculosis* identified in another specimen (e.g. gastric washing). Participants unable to provide sputa, or whose sputa samples were contaminated, were not considered to have newly-diagnosed TB; these participants contributed to the analysis. Participants who consented to enrolment (and by inference, consent to TB screening) but who did not consent to HIV testing were regarded not to have newly-diagnosed HIV infection; these participants contributed to the analysis. We adopted this strategy because we did not want to over-estimate the yield of SCI.

### Statistical analysis

We report the percentage yield of source case investigation as the number of newly-diagnosed TB disease cases identified by screening the contacts of every 100 children with TB. We defined the number needed to screen (NNS) to detect one case of TB as the number of persons screened divided by the number of persons with newly-diagnosed TB. The number needed to contact trace (NNCT) was defined as the number of childhood TB cases needed to identify one contact with newly-diagnosed TB by contact tracing: this was calculated as the number of childhood TB cases divided by number of contacts with newly diagnosed TB [7].

We examined differences in clinical and demographic characteristics between contacts with and without newly-diagnosed TB disease using t-tests (for continuous variables) and chi-square tests (for categorical variables). We performed univariate analysis, using generalized estimating equations (GEE) to account for household clustering, to identify factors associated with newly-diagnosed TB disease. Adjusted odds ratios (aORs) were calculated using a multivariable logistic GEE model with a household-level cluster term. Model variables included: age, education, employment status, smoking status, laboratory-confirmation of the sentinel case, TB symptoms, previous TB, HIV infection, household income, and room density. Statistical significance was defined as a p-value < 0·05. Data analysis was performed in R version 3.1.0 (The R Project for Statistical Computing).

## Results

### Characteristics of children with TB (Table 1)

We recruited 576 (45·7%) of 1261 children ≤7 years-of-age who were diagnosed with TB during their hospital admission over the study period. The median age of enrolled children was 11·5 months (interquartile range [IQR]: 5·9 to 26·3) and 47·7% were female. HIV infection was diagnosed in 248 (43·1%) children, and 129 (22·4%) children were HIV-exposed-but-uninfected (HEU). All HIV results in admitted children were confirmed by tests done as part of the routine hospital work up. Anthropometric data was available for 479 (83·2%) children, most of whom were malnourished: 57·8% were stunted, 16·1% were wasted and 25·9% severely wasted. Most children (n = 468; 81·2%) were diagnosed with pulmonary TB, but 108 (18·8%) had extra-pulmonary TB. Overall, 67 (11·6%) children had laboratory-confirmed TB, of whom 51 (8·9%) children had culture-confirmed TB. Multidrug-resistant *M*. *tuberculosis* was isolated from five children (i.e. 9·8% of all culture-confirmed TB).

**Table 1:** Demographic and clinical characteristics of children diagnosed with TB.

Age in months [median (IQR)]	11·5 (5·9 to 26·3)
Female gender [N (%)]	275 (47·7%)
Child’s HIV Status [N (%)]	
HIV-Infected	248 (43·1%)
HIV-Exposed but Uninfected	129 (22·4%)
HIV-Unexposed	191 (33·2%)
HIV-status undetermined	8 (1·4%)
Anthropometry	
Weight for age z score—WAZ [median (IQR)]	-2·53 (-3·91 to -1·31)
Height/Length for age z score—HAZ [median (IQR)]	-2·37 (-3·73 to -0·95)
Weight for height/length z score—WHZ [median (IQR)]	-1·44 (-2·93 to -0·08)
Stunting (HAZ<-2) [N (%)]	277 (57·8%)
Moderate Wasting (WHZ<-2) [N (%)]	77 (16·1%)
Severe Wasting (WHZ<-3) [N (%)]	124 (25·9%)
Type of Tuberculosis [N (%)]	
Pulmonary	468 (81·2%)
Extra-pulmonary	108 (18·8%)
Symptom duration [median, (IQR)]	
Duration of any symptoms in days	3 (2–7)
Duration of cough in days	4 (3–7)
Laboratory confirmation of TB [N (%)]	
Laboratory confirmed TB	67 (11·6%)
Non-laboratory confirmed TB	454 (78·8%)
No confirmatory investigations done	55 (9·5%)
Number of household contacts including caregiver (median, IQR)	4 (2 to 6)
Self-report of exposure to tuberculosis case at home [N (%)]	113 (19·7%)
Self-report of living with a person who is coughing [N (%)]	161 (28·1%)

Footnotes: Missing variables (>10 missing): WAZ– 67; HAZ and WHZ– 97 missing; Any symptoms– 48 missing; Cough duration– 169 missing; WAZ and WHZ were only calculated for children ≤5 years using the WHO growth standards. Most (90.6%) of the study children were ≤5 years.

### Characteristics of caregivers

**Fig 1:**
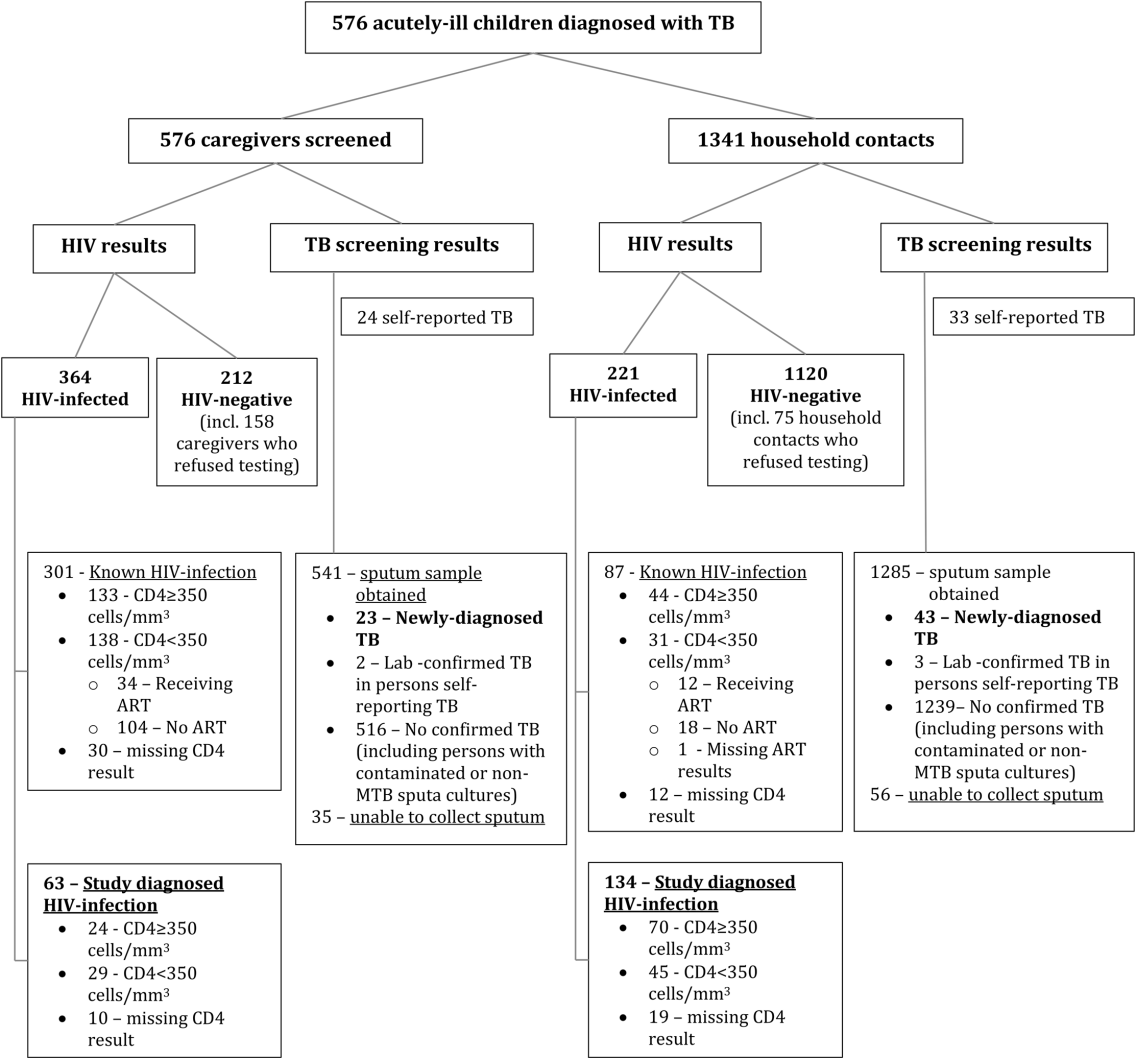
Source case investigation screening for tuberculosis and HIV infection in the caregivers and household contacts of hospitalised young children diagnosed with tuberculosis.

**Fig 2:**
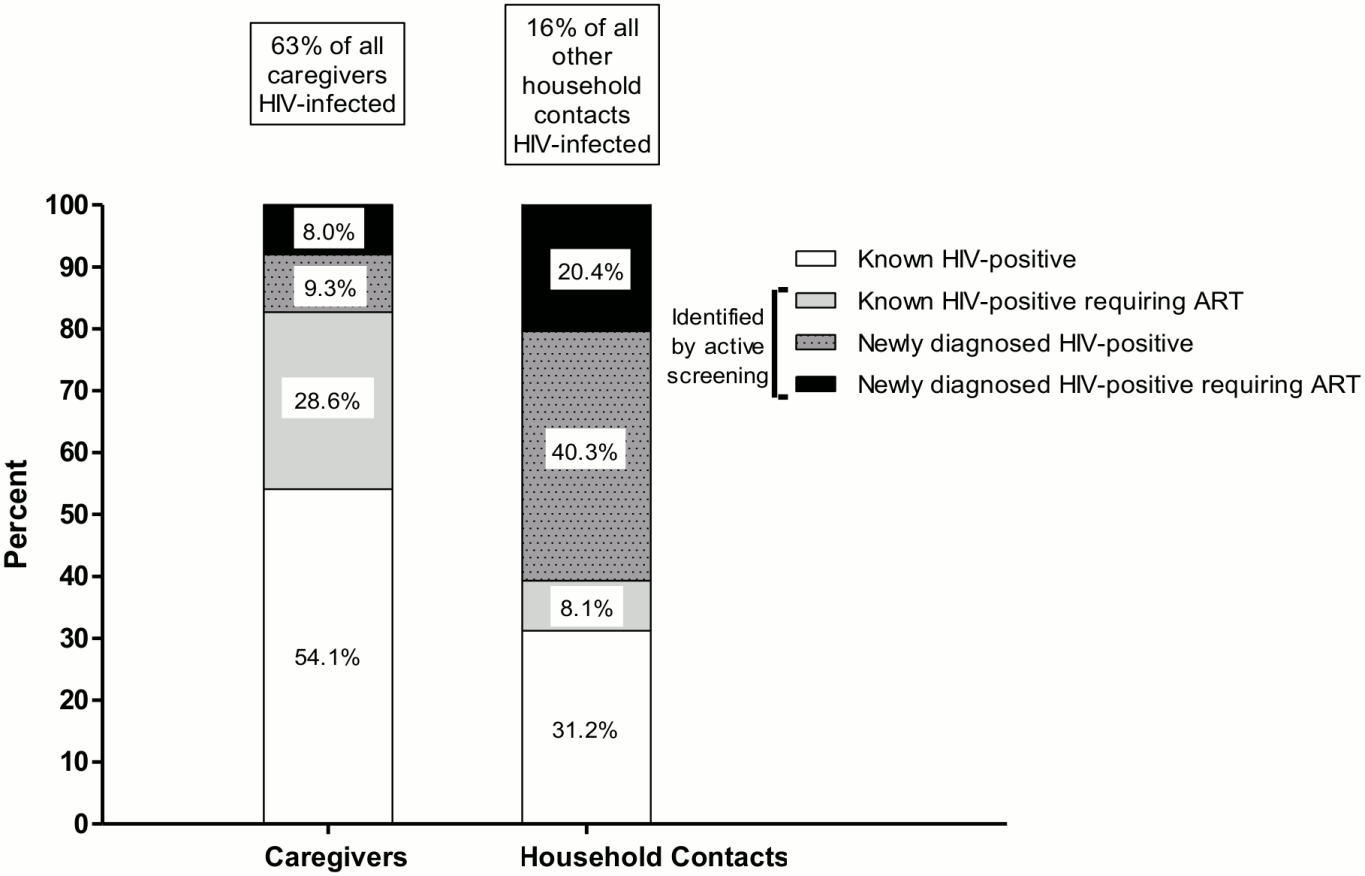
Source case investigation identifies newly-diagnosed HIV-infection and eligibility for ART in a significant proportion of caregivers and household contacts of hospitalised young children diagnosed with tuberculosis.

We screened 576 caregivers for TB and HIV. The median age of caregivers was 27·0 years (IQR: 23·0–33·0). Most (96·9%) caregivers were women, of whom 79·0% were the biologic mothers of sentinel children with TB (Table 2). Overall, 364 (63·2%) of 576 caregivers were HIV-infected; 301 (52·3%) self-reported to be HIV-infected at enrolment and 63 (10·9%) had HIV-infection detected during screening (Fig 1). The median CD4 cell count of HIV-infected caregivers was 342 cells/ mm^3^ (IQR: 196–547). Notably, 133 (79·6%) of 167 caregivers with CD4 <350 cells/ mm^3^ (i.e. eligible for ART according to South African guidelines [21]) were not receiving ART (Fig 2).

**Table 2:** Prevalence of HIV infection and TB-associated symptoms in caregivers with and without newly-diagnosed TB disease.

Variable	All caregivers (N = 576)	Caregivers with new TB disease (N = 23)	Caregivers with no new TB disease (N = 553)	p-value
Age in years [median (IQR)]	27·0 (23·0–33·0)	28·0 (23·5–33·8)	27·0 (23·0–33·0)	0·497
**TB-associated symptoms**				
Cough [N (%)]	84 (14·7)	10 (43·5)	74 (13·4)	**<0·001**
Cough ≥ 2 weeks [N (%)]	40 (7·0)	7 (30·4)	33 (6·0)	**<0·001**
Any TB symptom(s) [N (%)]	140 (24·4)	13 (56·5)	127 (23·1)	**<0·001**
Symptom duration in days [median (IQR)]	14·0 (7·0–33·5)	31·0 (7·0–46·0)	14·0 (7·0–29·75)	0·386
**TB status [N (%)]**				
Self-reported previous TB	45 (7·8)	4 (17·4)	41 (7·4)	0·096
Self-reported current TB	*24 (4·2)	^†^1 (4·8)	^‡^23 (4·2)	0·601
On TB therapy at enrolment	20 (3·5)	0 (0)	20 (3.6)	—
Laboratory-confirmed TB *(identified by screening)*	25 (4·6)	23 (100)	^§^2 (0·4)	—
**HIV Status [N (%)]**				
Known to be HIV infected	301 (52·3)	14 (60·9)	287 (51·9)	0·399
CD4 <350	138 (50·9)	8 (66·7)	130 (50·2)	0·378
Taking ARVs	74 (24·6)	3 (21·4)	71 (24·7)	>0·999
New HIV-infection *(identified by screening)*	63 (10·9)	4 (17·4)	59 (10·7)	0·302
CD4 <350	29 (54·7)	1 (25·0)	28 (57·1)	0·318
**Total HIV infection**	364 (63·2)	18 (78·3)	346 (62·6)	0·126

Footnotes: Any TB symptoms = Cough; Cough of 2 weeks or more; Coughing Blood; Fever; Weight Loss; Night Sweats; Loss of Appetite.

*24 caregivers self-reported TB.

^†^Of these, 1 caregiver did not access a health facility to have the diagnosis confirmed or to receive treatment; however, laboratory-confirmed TB was detected in this caregiver through study screening.

^‡^These 23 caregivers self-reported TB: 20 of these caregivers were receiving TB treatment

^§^Laboratory-confirmed TB was detected in 2 of the 20 caregivers receiving TB treatment. The remaining 3 caregivers who self-reported TB were not receiving TB treatment and did not have laboratory-confirmed TB detected through study screening.

Auramine microscopy and MGIT culture results were available for 541 (93·9%) caregivers able to expectorate a sputum sample, and newly-diagnosed TB disease was identified in 23 (4·0%) caregivers, six of whom were also AFB-positive. Laboratory-confirmed TB was also detected in two caregivers with self-reported TB who were currently receiving TB treatment. Another 24 (4·2%) caregivers reported current TB disease, 20 of whom were receiving concurrent TB treatment. Overall, 49 caregivers had TB, and 14 (56%) of 25 caregivers with culture-confirmed TB were HIV-co-infected.

### Other Household Contacts

We screened 1341 non-caregiver household contacts of whom 744 (55·5%) were female. The median age of household contacts was 16.0 years (IQR: 7.0 to 34.0) with a median of 3 contacts (IQR: 2-4) screened per household (Table 3). Overall, 221 (16·5%) household contacts were HIV-infected; the majority (n = 134; 60·6%) of whom were identified by source case investigation (Fig 1). The median CD4 count, available for 190 HIV-infected household contacts, was 422 cells/ mm^3^ (IQR: 244–621). Only 12 (15·8%) of 76 HIV-infected household contacts with CD4 ≤350 cells/ mm^3^ were receiving ART (Fig 2). Newly-diagnosed TB disease was identified in 43 (3·3%) of 1285 household contacts able to provide a sputum sample. Laboratory-confirmed TB was detected in three other non-caregiver household contacts with self-reported TB who were currently receiving TB therapy. Furthermore, another 33 contacts self-reported that they were currently receiving TB treatment. TB was detected in 13 (5·9%) of 221 HIV-infected household contacts, and in 33 (2·9%) of 1120 HIV-uninfected household contacts. Virtually all household contacts agreed to TB screening (1285 of 1341 produced a sputum sample) and 1045 of 1120 (93.3%) household contacts agreed to HIV testing.

**Table 3:** Prevalence of HIV infection and TB-associated symptoms in non-caregiver household members with and without newly-diagnosed TB disease.

Variable	All non-caregiver household contacts (N = 1341)	Non-caregiver household contacts with new TB disease (N = 43)	Non-caregiver household contacts with no new TB disease (N = 1298)	p-value
Age in years [median (IQR)]	16·0 (7·0–34·0)	14·0 (6·0–38·0)	16·0 (7·0–34·0)	0·701
**TB-associated symptoms**				
Cough [N (%)]	171 (12·8)	7 (16·3)	164 (12·7)	0·489
Cough ≥ 2 weeks [N (%)]	88 (6·6)	5 (11·6)	83 (6·4)	0·199
Any TB symptom(s) [N (%)]	237 (17·8)	10 (23·3)	227 (17·6)	0·337
Symptom duration in days [median (IQR)]	17·0 (7·0–38·0)	28·0 (9·0–32·0)	17·0 (7·0–38·0)	0·887
**TB status [N (%)]**				
Self-reported previous TB	72 (5·4)	5 (11·6)	67 (5·2)	0·076
Self-reported current TB	*33 (2·5)	0 (0)	33 (2·6)	0·622
On TB treatment at enrolment	27 (2·0)	0 (0)	^†^27 (2·1)	—
Laboratory-confirmed TB *(identified by screening)*	46 (3·4)	43 (100)	^‡^3 (0·2)	—
**HIV Status [N (%)]**				
Known to be HIV infected	87 (6·5)	6 (14·0)	81 (68·7)	0·055
CD4<350	31 (41·3)	4 (80·0)	27 (38·6)	0·153
Taking ARVs	44 (51·2)	4 (66·7)	40 (50·0)	0·677
New HIV-infection *(identified by screening)*	134 (10·0)	7 (16·3)	127 (9·8)	0·189
CD4<350	45 (39·1)	4 (57·1)	41 (38·0)	0·426
**Total HIV infection**	221 (16·5)	13 (30·2)	208 (16·0)	0·014

Footnotes: Any TB symptoms = Cough; Cough of 2 weeks or more; Coughing Blood; Fever; Weight Loss; Night Sweats; Loss of Appetite.

*33 non-caregiver household contacts self-reported TB:

^†^27 of these non-caregiver household contacts were receiving TB treatment

^‡^Laboratory-confirmed TB was detected in 3 of these 27 non-caregiver household contacts who were receiving TB treatment. The remaining 6 non-caregiver household contacts who self-reported TB were not receiving TB treatment and did not have laboratory-confirmed TB detected through study screening.

Overall, we detected newly-diagnosed HIV infection and newly-diagnosed TB disease in 10·3% (95% CI: 9.0–11.7%) and 3·4% (95% CI: 2.7–4.4%) of all close contacts of a sentinel TB case (Table 4). The HIV detection rate increases to 11.7% if the yield is calculated in only those close contacts who actually underwent HIV testing (n = 1684). A caregiver or non-caregiver household contact with either newly-diagnosed TB disease or self-reported TB was detected in 100 (17·4%) of all households.

**Table 4:** Prevalence of HIV infection and TB-associated symptoms in all household contacts (caregivers and non-caregiver household contacts) with and without newly diagnosed TB disease.

Variable	All household contacts (Caregivers and non-caregiver household contacts) (N = 1917)	All household contacts with new TB disease (N = 66)	All household contacts with no New TB disease (N = 1852)	p-value
Age in years [median (IQR)]	23·0 (10·0–33·0)	26·0 (11·0–35·0)	23·0 (10·0–33·0)	0·491
**TB-associated symptoms**				
Cough [N (%)]	255 (13·4)	17 (25·8)	238 (12·9)	0·003
Cough ≥ 2 weeks [N (%)]	128 (6·7)	12 (18·2)	116 (6·3)	0·001
Any TB symptom(s) [N (%)]	377 (19·8)	23 (34·9)	354 (19·2)	0·002
Duration of symptoms in days [median (IQR)]	15·0 (7·0–35·5)	28·5 (7·5–43·5)	15·0 (7·0–35·0)	0·750
**TB status [N (%)]**				
Self-reported previous TB	117 (6·1)	9 (13·6)	108 (5·8)	0·017
Self-reported current TB	*57 (3·0)	^†^1 (1·5)	^‡^56 (3·0)	>0·999
On TB treatment at enrolment	47 (2·5)	0 (0)	47 (2·5)	—
Laboratory-confirmed TB *(identified by screening)*	71 (3·8)	66 (100)	^§^5 (0·3)	—
**HIV Status**				
Known to be HIV infected	388 (20·3)	20 (30·3)	368 (19·9)	0·039
CD4<350	169 (48·8)	12 (70·6)	157 (47·7)	0·066
Taking ARVs	118 (30·5)	7 (35·0)	111 (30·3)	0·653
New HIV-infection *(identified by screening)*	197 (10·3)	11 (16·7)	186 (10·1)	0·082
CD4<350	74 (44·1)	5 (45·5)	69 (44·0)	>0·999
**Total HIV infection**	585 (30·5)	31 (47·0)	554 (30·0)	0·003

Footnotes: Any TB symptoms = Cough; Cough of 2 weeks or more; Coughing Blood; Fever; Weight Loss; Night Sweats; Loss of Appetite.

*57 household contacts self-reported TB.

^†^Of these, 1 caregiver did not access a health facility to have the diagnosis confirmed or to receive treatment; however, laboratory-confirmed TB was detected in this caregiver through study screening.

^‡^In the remaining 56 household contacts who self-reported TB, 47 were also receiving TB treatment

^§^Laboratory-confirmed TB was detected in 5 of the 47 household contacts receiving TB treatment. The remaining 9 household contacts who self-reported TB were not receiving TB treatment and did not have laboratory-confirmed TB detected through study screening.

### Predictors of newly-diagnosed TB disease in caregivers and household contacts

Univariate and multivariate analyses identifying variables that increased the likelihood of detecting newly-diagnosed TB in caregivers and other household contacts are described in S1–S3 Tables. In summary, multivariate analysis suggested that the presence of TB symptoms was the main risk factor for detecting newly-diagnosed TB in caregivers (adjusted OR: 5·07; 95% CI: 2·09 to 12·33). While caregiver HIV infection increased the odds of newly-diagnosed TB disease in caregivers, this association was not statistically significant (adjusted OR: 2·89; 95% CI: 0·91 to 9·11). Laboratory-confirmed TB in the sentinel paediatric case was not associated with newly-diagnosed TB disease in the caregiver. Among non-caregiver household contacts, multivariate analysis suggested that HIV-infection in the caregiver as well as household conditions predisposing to air-borne transmission of pathogens (i.e. the number of individuals per window or door) were the main risk factors for detecting newly-diagnosed TB disease in household contacts (adjusted OR: 2·80; 95% CI: 1·21 to 6·46 and 1·29, 95% CI: 1·05 to 1·59 respectively).

Overall, among all close contacts, multivariate analysis suggested that two factors increased the likelihood of detecting newly-diagnosed TB disease: (i) the presence TB symptoms in either caregivers or other household contacts (adjusted OR: 2·01; 95% CI: 1·11 to 3·62) and (ii) HIV-infection in either caregivers or other household contacts (adjusted OR: 2·16; 95% CI: 1·20 to 3·90).

### The effect of contact tracing targeted to caregivers who report TB symptoms and/or are HIV-infected

There were 405 (70·3%) caregivers who were either HIV-infected or who reported TB symptoms. If our source case investigation programme targeted individuals reporting TB symptoms and/or those who are HIV-infected, then the detection rates of newly-diagnosed TB disease would increase to: (i) 12·1% in HIV-infected caregivers reporting TB symptoms (n = 99); (ii) 9·3% in all caregivers reporting TB symptoms (n = 140); and (iii) 4·9% in all HIV-infected caregivers (n = 364). Although targeted screening would have increased detection rates, newly-diagnosed TB disease would be missed in four of 171 (2·3%) caregivers who were HIV-uninfected or asymptomatic [S4 Table and S1 Fig].

### Number needed to screen (NNS) and number needed to contact trace (NNCT) (Table 5)

Overall, 29 close contacts required screening to detect one contact with newly-diagnosed TB disease (i.e. NNS); screening the close contacts of every nine children diagnosed with TB identified one contact with newly-diagnosed TB disease (i.e. NNCT). Ten close contacts required screening to detect one case of newly-diagnosed HIV infection (i.e. NNS); screening the close contacts of every three children would yield one contact with HIV infection (i.e. NNCT). Similarly, one person requiring ART was identified from every ten close contacts screened (i.e. NNS); screening the close contacts of every three young children would identify one person requiring ART (i.e. NNCT). In summary, screening all the close contacts of every nine children diagnosed with TB would identify one case of newly-diagnosed TB disease, three cases of newly-diagnosed HIV-infection, and three HIV-infected persons eligible for ART.

**Table 5:** The yield of contact tracing from source case investigation of children with sentinel TB: The number needed to screen* (NNS) and the number needed to contact trace^†^ (NNCT).

	Caregivers	Household contacts	Total
**New TB:** NNS	25 (576/23)	32 (1341/43)	29 (1917/66)
**New TB:** NNCT	25 (576/23)	32 (576/43)	9 (576/66)
**New HIV:** NNS	10 (576/63)	10 (1341/134)	10 (1917/197)
**New HIV:** NNCT	10 (576/63)	5 (576/134)	3 (576/197)
**ART:** NNS	5 (576/133)	22 (1341/63)	10 (1917/196)
**ART:** NNCT	5 (576/133)	10 (576/63)	3 (576/196)

Footnote:

*The minimal number of caregivers and household contacts that require screening (NNS) to identify one person with newly-diagnosed TB disease, HIV infection, or one HIV-infected person who requires immediate ART.

^†^The number needed to contact trace (NNCT) refers to the minimum number of index children needed to initiate contact tracing to identify one person with newly-diagnosed TB disease, HIV infection, or one HIV-infected person who requires immediate ART.

## Discussion

We found an extremely high prevalence of newly-diagnosed HIV-infection and a high prevalence of newly-diagnosed TB disease in the caregivers of young children diagnosed with TB during their hospital admission for an acute illness. An important study finding was the detection of HIV infection in almost one quarter of caregivers who self-reported a negative or unknown HIV-status, and who also consented to HIV testing. This is concerning because most caregivers ought to have recently accessed PMTCT programmes that offer HIV testing [22–24]. In our study, an unexpectedly high proportion (79·6%) of HIV-infected caregivers were not receiving ART despite having CD4 counts≤350 cells/mm^3^; the reasons for this need to be determined because provision of ART to mothers improves both maternal and child survival [25, 26].

Despite the inherent difficulty in diagnosing childhood TB and knowing that the contacts’ HIV and TB status is likely to have influenced the clinicians’ decision to diagnose TB in children in the first instance, our results suggest that integrated source case investigation is a valuable activity. The high TB detection rate in our contacts self-reporting negative or unknown TB infection (3·5%) is comparable to TB detection rates found in other studies conducted in high burden settings [2, 27] and is higher than the corresponding rate for newly-diagnosed TB disease (1·7%) reported in our study district during the study period [28]. Screening caregivers at the hospital is especially important: this screening is convenient and relatively inexpensive, and targeting caregivers who report TB symptoms and/or are HIV-infected identifies those who are at even greater risk (4·9–12·1%) of having newly-diagnosed TB disease.

However, our results suggest that young children may also acquire TB infection from extra-household sources such as crèches or day-care centres: we only found individuals with self-reported or newly-diagnosed TB in 8 of 67 (11·9%) households accommodating children with laboratory-confirmed TB.

Our TB detection rates are higher than the rates described in other studies that have evaluated the use of source case investigation [4–8]. Our study therefore lends further support to the use of source case investigation, and underscores the importance of integrating TB and HIV screening, including a determination of ART eligibility in HIV-infected contacts.

This observational study has several limitations. There is potential selection bias because we only recruited children whose caregivers were present in the wards during working hours and there may be differences in HIV and TB prevalence in those caregivers who were unavailable during these times. Secondly, we did not determine whether those contacts requiring TB treatment or ART actually accessed care, which is essential to ascertain both individual benefit and cost-effectiveness of source case investigation. In addition, we did not collect detailed data on caregivers’ experiences of PMTCT programmes—this is especially important because the prevalence of HIV-infection is falling dramatically in young infants at our institution [S6 File] as more effective PMTCT interventions are implemented. Finally, some laboratory results, especially those relating to confirmation of *M tuberculosis* disease in hospitalised children, and CD4^+^ results in HIV-infected close contacts, were missing. We may have diagnosed more cases of TB if we collected two sputum samples (including an early morning or overnight sample) but we could not do so for logistic reasons. We may also have missed TB and HIV infection in those household contacts who declined study participation and HIV testing. The number of caregivers who refused HIV testing is concerning, and measures designed to increase HIV testing uptake in caregivers are required. In addition, because we regarded missing sputum samples and missing CD4^+^ counts in HIV-infected individuals as negative results for TB and for ART initiation eligibility, our estimates represent conservative rates of newly-diagnosed TB disease and the minimal proportion of HIV-infected individuals eligible for ART.

In addition to missing data, the reasons for diagnosing TB were not always systematically documented in the clinical notes. Many clinicians do not necessarily use algorithms to diagnose childhood TB because almost all current diagnostic algorithms [29] fail to recognise that TB can present as an acute illness in young children [14, 15, 20]. At our institution, about 11% of all acutely-ill children with culture-confirmed bacterial pneumonia have concurrent culture-proven MTB infection [20], and these children would not be treated for TB if current diagnostic algorithms were relied upon. However, this practice makes it likely that the attending paediatricians erred towards over-diagnosing childhood TB, and some of our study children may have had non-TB pneumonia. We also did not compare the effectiveness of integrated HIV and TB screening in a control group (i.e. the close contacts of young children diagnosed with acute non-TB pneumonia, or the close contacts of admitted children with non-respiratory diseases). Nonetheless, these limitations should not detract from the value of source case investigation given the high yields of TB and HIV detection.

## Conclusion

In conclusion, our study suggests that source case investigation yields high rates of previously undiagnosed HIV and TB infection in close contacts (especially the caregiver) of young children who have been diagnosed with TB during hospital admission for an acute illness. Further controlled studies, incorporating costing analyses and using a new TB diagnostic algorithm that is specifically adapted for use in acutely-ill children with short symptom duration, should evaluate the use of integrated source case investigation in high burden countries—these programmes should be implemented if our findings are confirmed.

## Supporting Information

S1 Fig A proportional Venn diagram showing the relationship between the prevalence of newly diagnosed TB disease, HIV infection, and the presence of TB symptoms in the caregivers of young children diagnosed with TB. The relationship between the prevalence of newly diagnosed TB disease (red circle), HIV infection (orange circle), and the presence of TB symptoms (blue circle) in the caregivers (n = 576) of young children diagnosed with TB. *Note: The presence or absence of TB symptoms was not recorded in three caregivers—they are assumed to be asymptomatic.
(TIF)

S1 File Materials and Methods: Participants. This supplementary file provides information about the diagnosis of tuberculosis in young children.
(DOCX)

S2 File Source Case Investigation—Index child interview. This questionnaire was used to collect information about the index child’s TB symptoms.
(PDF)

S3 File Source Case Investigation—Caregiver interview. This questionnaire was used to collect information about the caregiver’s TB symptoms.
(PDF)

S4 File Source Case Investigation—Other household contact interview. This questionnaire was used to collect information about household contacts’ TB symptoms.
(PDF)

S5 File Source Case Investigation—Household information. This questionnaire was used to collect information about the physical structure of the household dwelling.
(PDF)

S6 File The prevalence of malnutrition in children admitted to a general paediatric ward at the Chris Hani Baragwanath Academic Hospital: A cross-sectional survey. This paper describes the decreasing prevalence of HIV infection in young infants admitted to the Chris Hani Baragwanath Academic Hospital.
(PDF)

S1 Table Predictors of newly diagnosed TB disease among caregivers of children with TB. (DOCX)

S2 Table Predictors of newly diagnosed TB disease among non-caregiver household contacts of children with TB. (DOCX)

S3 Table Predictors of newly diagnosed TB disease among all contacts (caregivers and non-caregiver household members) (DOCX)

S4 Table The effect of targeted screening on the yield of newly diagnosed TB in caregivers who report TB symptoms and/or are HIV infected. (DOCX)
