# Supplementary material for: Integrated Source Case Investigation for Tuberculosis (TB) and HIV in the Caregivers and Household Contacts of Hospitalised Young Children Diagnosed with TB in South Africa: An Observational Study
Source: PLoS One. 2015 Sep 17;10(9):e0137518. doi: 10.1371/journal.pone.0137518 (PMC4574562; doi:10.1371/journal.pone.0137518)
Supplement: S1 File — This supplementary file provides information about the diagnosis of tuberculosis in young children. (DOCX) [file pone.0137518.s002.docx]

**Materials and Methods**

**Participants:** We recruited the primary caregivers and household contacts of children <7 years who were diagnosed with TB during an admission for an acute illness to the Chris Hani Baragwanath Academic Hospital (CHBAH) in Soweto, South Africa. In this study, we approached the caregivers for possible enrolment after the diagnosis of TB was made by the attending paediatrician. At this hospital, clinicians consider factors such as symptoms (i.e. cough, fever, and/or weight loss), history of close contact with a TB case, chest X ray findings, tuberculin skin tests, and other relevant tests (such as lumbar punctures, CT scans and lymph node biopsies) when making decisions to start TB treatment in children [1]. The clinicians do not, however, specifically grade the children as having ‘highly probable’, ‘probable’ or ‘possible’ TB (as defined by Nicol [2]). Although gastric washings (for auramine-stained microscopy and culture) were collected from most children, TB treatment was started in advance of receipt of the final microbiological result in most cases. We defined children whose gastric aspirates were positive for acid-fast bacilli on smear microscopy, and/or had cultures positive for *Mycobacterium tuberculosis* on liquid culture, or who had histological features typical of TB (e.g. lymph node biopsy), as having laboratory-confirmed TB.

**References:**

1. Newton SM, Brent AJ, Anderson S, Whittaker E, Kampmann B. Paediatric tuberculosis. Lancet Infect Dis. 2008;8:498-510.
2. Nicol MP, Workman L, Isaacs W, et al. Accuracy of the Xpert MTB/RIF test for the diagnosis of pulmonary tuberculosis in children admitted to hospital in Cape Town, South Africa: a descriptive study. Lancet Infect Dis. 2011;11:819-824
