# Supplementary material for: Integrated Source Case Investigation for Tuberculosis (TB) and HIV in the Caregivers and Household Contacts of Hospitalised Young Children Diagnosed with TB in South Africa: An Observational Study
Source: PLoS One. 2015 Sep 17;10(9):e0137518. doi: 10.1371/journal.pone.0137518 (PMC4574562; doi:10.1371/journal.pone.0137518)
Supplement: S2 File — This questionnaire was used to collect information about the index child’s TB symptoms. (PDF) [file pone.0137518.s003.pdf]

ACF-Kids (063) Plt 1 (001) Visit 1 (010)

Index ID ☐☐☐☐ 0 1

Index Child's Interview

Interview Date ☐☐ (dd) ☐☐☐ (MMM) 2 0 1 ☐ (yyyy)

Interviewer Initials: ☐☐

Consent obtained from caregiver?........ ☐ Yes ☐ No

Index Case Details:

1. Sex:......................... ☐ Male ☐ Female
2. Date of birth:........... ☐☐ ☐☐☐ 2 0 ☐☐ *(dd/MMM/yyyy)*
3. Child is:................... ☐ Outpatient ☐ Inpatient

Sociodemographic Information:

4. How many years has the child lived in this house?.................... ☐☐.☐☐ yrs
5. How many people reside in the child's house?.......................... ☐☐
6. Estimate of combined household income of the child's house?... ☐☐☐☐☐☐ R/mth
7. Are there any smokers in the child's house?.............................. ☐ Yes ☐ No
   7a. If Yes to Q7, do they smoke indoors?................................. ☐ Yes ☐ No
8. Are there any person(s) living in the child's house that have been to prison? ☐ Yes ☐ No → ***If No, go to Q9***
   8a. How many months did that person spend in prison?............................. ☐☐☐
   8b. When was the person last in prison?.. ☐☐☐ (MMM) ☐☐☐☐ (yyyy)
   8c. Is that person coughing now?... ☐ Yes ☐ No
   8d. Has that person been diagnosed with TB?... ☐ Yes ☐ No ☐ Don't know

9. Does anyone in the child's house currently have TB, or had TB in the past two years?.. ☐ Yes ☐ No → ***If No, go to Q10***

   If Yes to Q9, how many people?.......... ☐☐
   (Complete relevant lines below)

a.	Age ☐☐ →	Sex ☐ M ☐ F →	Number of previous TB episodes before 2008 ☐
b.	Age ☐☐ →	Sex ☐ M ☐ F →	Number of previous TB episodes before 2008 ☐
c.	Age ☐☐ →	Sex ☐ M ☐ F →	Number of previous TB episodes before 2008 ☐
d.	Age ☐☐ →	Sex ☐ M ☐ F →	Number of previous TB episodes before 2008 ☐

10. Index child's height/length:.... ☐☐☐.☐ cm
11. Index child's weight:............. ☐☐.☐ kg

☐☐☐ Child's Initials

Staff Initials / Date

Active Case Findings - ACF Kids

Please Initial and date the appropriate section below:

1st Review:	____________ Initials	_____/_____/20_____ Date	*Faxed by:* ____________ Initials	_____/_____/20_____ Date
2nd Review:	____________ Initials	_____/_____/20_____ Date	*Faxed by:* ____________ Initials	_____/_____/20_____ Date
3rd Review:	____________ Initials	_____/_____/20_____ Date	*Faxed by:* ____________ Initials	_____/_____/20_____ Date
4th Review:	____________ Initials	_____/_____/20_____ Date	*Faxed by:* ____________ Initials	_____/_____/20_____ Date

Index ID [| | |] [0|1]

Index Child's Interview

History of current illness:

12. What was the start date of the symptoms that brought the child to hospital?........ dd [|] MMM [| |] yyyy [2|0|1|]

13. What are Index child's current symptoms?...

	Yes	No		
Cough	☐	☐	If yes, duration:	[| |] days
Weight loss	☐	☐	If yes, duration:	[| |] days
Fever	☐	☐	If yes, duration:	[| |] days
Lethargy	☐	☐	If yes, duration:	[| |] days
Night sweats	☐	☐	If yes, duration:	[| |] days
Loss of appetite	☐	☐	If yes, duration:	[| |] days

TB History and risk:

14. Has the child had TB previously?........ Yes ☐ No ☐ → ***If No, go to Q17.***
    If Yes, number of episodes?........ [ ]

15. What was the date of diagnosis of your child's most recent TB episode?........ MMM [| |] yyyy [2|0| |]
    (Before current admission)

16. Did child complete full TB treatment?........ Yes ☐ No ☐

17. Is there any person living with this child who is coughing?........ Yes ☐ No ☐
    If Yes, how long has that person been coughing? [|] wks

	dd	MMM	yyyy
18. Date of admission of the child?........	[|]	[| |]	[2|0|1|]
19. Date of discharge or death of the child?........	[|]	[| |]	[2|0|1|]

20. Diagnosis (ICD-10)?........
    Ask Dr Moor or Dr Lala for assistance and draw a line through the UNUSED diagnoses fields.

a. [ ] [|].[ ]
b. [ ] [|].[ ]
c. [ ] [|].[ ]
d. [ ] [|].[ ]
e. [ ] [|].[ ]

☐ Check this box if child died in hospital.

21. HIV status of mother at the time of this child's birth?........ ☐ HIV infected ☐ HIV uninfected ☐ HIV unknown

22. HIV status of this child?........ ☐ HIV infected ☐ HIV uninfected

22a. Child receiving Antiretroviral therapy?........ ☐ Yes ☐ No

i. Date ARVs started?...... MMM [| |] yyyy [2|0| |]

ii. List ARV codes below:

a. [|] b. [|] c. [|]
d. [|] e. [|] f. [|]

Active Case Findings - ACF Kids

Please Initial and date the appropriate section below:

1st Review: ____________	______/______/20______	*Faxed by:* ____________	______/______/20______
Initials	Date	Initials	Date

2nd Review: ____________	______/______/20______	*Faxed by:* ____________	______/______/20______
Initials	Date	Initials	Date

3rd Review: ____________	______/______/20______	*Faxed by:* ____________	______/______/20______
Initials	Date	Initials	Date

4th Review: ____________	______/______/20______	*Faxed by:* ____________	______/______/20______
Initials	Date	Initials	Date

Index ID [ ][ ][ ][ ] [0][1]

Index Child's Interview

23. Child's chest X-ray?............... ☐ Done ☐ Not done → ☐ Normal ☐ Abnormal

☐ Adenopathy ☐ Infiltrate/Consolidation ☐ Miliary pattern
☐ Pleural effusion ☐ Cavitation ☐ Other: ____________________

24. Abdominal sonar?.................... ☐ Not done ☐ Normal ☐ Splenic microabscesses
☐ Lymphadenopathy ☐ Other: ____________________

25. Tuberculosis skin test (TST/PPD) at this admission?..... [ ][ ] mm *(99 if not done)*

26. Date sputum specimen collected:................................. [ ][ ] [ ][ ][ ] [2][0][1][ ] *(dd/MMM/yyyy)*

27. Sputum smear (AFB):.. ☐ Done ☐ Not done
(If any positive, use the highest count.)

Result: ☐ Negative/Not seen ☐ Moderate (++) ☐ Unknown
☐ Scanty (+) ☐ Numerous (+++)

28. Culture:... ☐ Done ☐ Not done

28a. Result: ZN for AAFB:................................. ☐ Positive ☐ Negative ☐ Unknown

28b. Number of days to positive:......... [ ][ ]

28c. ID of Mycobacterium:................... ☐ M Tuberculosis ☐ Other
☐ M Avium Complex ☐ Contaminated / Lab problem

28d. Drug sensitivity:....................................... ☐ Done ☐ Not Done

Isoniazid: ☐ Resistant ☐ Sensitive ☐ Not done
Rifampin: ☐ Resistant ☐ Sensitive ☐ Not done

If resistant to Isoniazid AND Rifampacin →

Resistant to 2nd line drugs?..................... ☐ Yes ☐ No
(If Yes, specify which drugs below)

a. ____________________

b. ____________________

29. Other biological specimen:.............................. ☐ Collected ☐ Not collected
(e.g. FNA, biopsy, gastric aspirate)

29a. Specify: ____________________

29b. Date obtained:. [ ][ ] [ ][ ][ ] [2][0][1][ ]
dd MMM yyyy

29c. Result:............ ☐ TB ☐ Not TB ☐ Unknown

Active Case Findings - ACF Kids

Please Initial and date the appropriate section below:

1st Review:	____________ Initials	_____/_____/20_____ Date	*Faxed by:*	____________ Initials	_____/_____/20_____ Date
2nd Review:	____________ Initials	_____/_____/20_____ Date	*Faxed by:*	____________ Initials	_____/_____/20_____ Date
3rd Review:	____________ Initials	_____/_____/20_____ Date	*Faxed by:*	____________ Initials	_____/_____/20_____ Date
4th Review:	____________ Initials	_____/_____/20_____ Date	*Faxed by:*	____________ Initials	_____/_____/20_____ Date

Index ID ☐☐☐☐ 0 1

Index Child's Interview

30. Child on TB treatment?........... ☐ Yes ☐ No

30a. Date TB treatment started?.. dd ☐☐ MMM ☐☐☐ yyyy 2 0 1 ☐

30b. Regimen?............................
- ☐ 1st episode
- ☐ 2nd episode
- ☐ TBM
- ☐ MDR/XDR
- ☐ "Liver friendly"

31. Pyridoxine given to child?......... ☐ Yes ☐ No

32. Prednisone given to child?....... ☐ Yes ☐ No

Active Case Findings - ACF Kids

Please Initial and date the appropriate section below:

1st Review:	____________ Initials	_____/_____/20_____ Date	*Faxed by:*	____________ Initials	_____/_____/20_____ Date
2nd Review:	____________ Initials	_____/_____/20_____ Date	*Faxed by:*	____________ Initials	_____/_____/20_____ Date
3rd Review:	____________ Initials	_____/_____/20_____ Date	*Faxed by:*	____________ Initials	_____/_____/20_____ Date
4th Review:	____________ Initials	_____/_____/20_____ Date	*Faxed by:*	____________ Initials	_____/_____/20_____ Date
