# Supplementary material for: Integrated Source Case Investigation for Tuberculosis (TB) and HIV in the Caregivers and Household Contacts of Hospitalised Young Children Diagnosed with TB in South Africa: An Observational Study
Source: PLoS One. 2015 Sep 17;10(9):e0137518. doi: 10.1371/journal.pone.0137518 (PMC4574562; doi:10.1371/journal.pone.0137518)
Supplement: S3 File — This questionnaire was used to collect information about the caregiver’s TB symptoms. (PDF) [file pone.0137518.s004.pdf]

ACF-Kids (063) Plt 12 (012) Visit 1 (010)

Household ID [ ][ ][ ][ ] Study ID [0][2]

Use ID number from HH census

Child's Caregiver's Information

Interview Date [ ][ ] dd [ ][ ][ ] MMM [2][0][1][ ] yyyy

This form should be completed for every consenting or assenting HH member

Instructions to interviewer:

Was a consent form signed *(or assent if child)*....... ☐ Yes ☐ No → ***If No, STOP and do consent***

Demographic information

1. Caregiver's sex:................ ☐ Male ☐ Female

2. Caregiver's date of birth:... [ ][ ][ ] MMM [ ][ ][ ][ ] yyyy **OR**, if exact date of birth not known, estimated age:.................................. [ ][ ] yrs

3. Caregiver's initials *(first/middle/last)*.. [ ][ ][ ] First/Middle/Last

4. How long have you been living in your house?.................................... [ ][ ].[ ][ ] yrs

5. About how many hours per day do you spend inside your house?.......
   a. Weekdays:...... [ ][ ] hrs
   b. Weekends:...... [ ][ ] hrs

6. Highest grade of education you attended:.. [ ][ ] →
   00 = No education
   01-12 = Highest grade attended
   13 = Any post school non degree/diploma
   14 = Degree/Diploma

7. Caregiver's occupational status:
   ☐ Self-employed
   ☐ Student
   ☐ Salaried worker
   ☐ Other,specify: ____________________
   ☐ Unemployed but able to work
   ☐ Unemployed and unable to work
   ☐ Occasional work (piece Jobs)

8. Sputum specimen taken from caregiver?.. ☐ Yes ☐ No

   8a. Did you induce a specimen?............. ☐ Yes ☐ No

   8b. Specimen bar code number?............ [ ][ ][ ][ ][ ][ ][ ][ ]

   8b. Date specimen taken:....................... [ ][ ] dd [ ][ ][ ] MMM [2][0][1][ ] yyyy

Staff Initials / Date

Active Case Findings - ACF Kids

Please Initial and date the appropriate section below:

1st Review: ____________	______/______/20______	*Faxed by:* ____________	______/______/20______
Initials	Date	Initials	Date
2nd Review: ____________	______/______/20______	*Faxed by:* ____________	______/______/20______
Initials	Date	Initials	Date
3rd Review: ____________	______/______/20______	*Faxed by:* ____________	______/______/20______
Initials	Date	Initials	Date
4th Review: ____________	______/______/20______	*Faxed by:* ____________	______/______/20______
Initials	Date	Initials	Date

Household ID [ ][ ][ ][ ] Study ID 0 2

Child's Caregiver's Information

9. Do you currently have TB?.................... ☐ Yes (Go to Q9a) ☐ No ☐ Don't know (Go to Q9e)

9a. Date of diagnosis... [ ][ ] dd [ ][ ][ ] MMM 2 0 [ ][ ] yyyy

9b. Do you have a TB treatment card?... ☐ Yes ☐ No

9c. Treatment card visualised?.............. ☐ Yes ☐ No

9d. Treatment status?........................... ☐ Currently on treatment ☐ Interrupted/Defaulted *Have not completed ≥6 months of TB treatment*

→ *(Go to TB History and Risk.)*

9e. Symptoms present at visit. *(Ask about each symptom separately.)*

	No	Yes	Duration in days
9e1. Cough................................	☐	☐ →	[ ][ ][ ]
9e2. Coughing sputum................	☐	☐ →	[ ][ ][ ]
9e3. Coughing blood..................	☐	☐ →	[ ][ ][ ]
9e4. Weight loss........................	☐	☐ →	[ ][ ][ ]
9e5. Fever.................................	☐	☐ →	[ ][ ][ ]
9e6. Soaking night sweats..........	☐	☐ →	[ ][ ][ ]
9e7. Shortness of breath............	☐	☐ →	[ ][ ][ ]
9e8. Loss of appetite.................	☐	☐ →	[ ][ ][ ]

9f. Date of first onset of these symptoms:.................................... [ ][ ] dd [ ][ ][ ] MMM 2 0 [ ][ ] yyyy

9g. If any TB symptoms present:

9g1. Date first sought medical help for these TB symptoms:... [ ][ ] dd [ ][ ][ ] MMM 2 0 [ ][ ] yyyy

OR: Check this box if did not seek medical help:.......... ☐

9g2. Number of visits made for medical help for these symptoms:... [ ][ ]

9g2a. Type of medical help sought: *(Tick all that apply)*

☐ Hospital ☐ Primary care clinic ☐ Pharmacist

☐ TB clinic ☐ VCT centre ☐ Traditional healer

☐ Other, specify: ____________________

Active Case Findings - ACF Kids

Please Initial and date the appropriate section below:

1st Review: ____________ Initials	______/______/20______ Date	*Faxed by:* ____________ Initials	______/______/20______ Date
2nd Review: ____________ Initials	______/______/20______ Date	*Faxed by:* ____________ Initials	______/______/20______ Date
3rd Review: ____________ Initials	______/______/20______ Date	*Faxed by:* ____________ Initials	______/______/20______ Date
4th Review: ____________ Initials	______/______/20______ Date	*Faxed by:* ____________ Initials	______/______/20______ Date

Household ID [ ][ ][ ][ ] Study ID [0][2]

Child's Caregiver's Information

1. Did you ever have TB before in your life?.................................. ☐ Yes ☐ No
   - Yes → ***Go to Q1a***
   - No → ***Go to Q2***

   1a. What was the date of your most recent TB diagnosis?...... MMM [ ][ ][ ] yyyy [ ][ ][ ][ ]

   1b. Did you complete the full TB treatment ?........................... ☐ Yes ☐ No

   1c. How many months did you take TB treatment for?............ [ ][ ]

2. Do you currently smoke tobacco?.. ☐ Yes ☐ No
   - Yes → ***Go to Q2a***
   - No → ***Go to Q3***

   2a. How many cigarettes or pipes per day?........................... [ ][ ]

   2b. When did you start?... [ ][ ][ ][ ] year

   2c. Do you smoke inside your home?...................................... ☐ Yes ☐ No

HIV information

3. Have you previously tested **positive** for HIV?.......................... ☐ Yes ☐ No
   - Yes → ***Go to Q3a***
   - No → ***Go to Household HIV testing***

   3a. What was the date?... dd [ ][ ] MMM [ ][ ][ ] yyyy [ ][ ][ ][ ]

   3b. Are you currently taking ARVs?.. ☐ Yes ☐ No

Household HIV testing for this person

4. Consent for VCT?.. ☐ Yes ☐ No → ***Go to Q4b***

   4a. Rapid test results:
   - ☐ 2 Positive Rapids → Blood drawn for CD4 count?............. ☐ Yes ☐ No
   - ☐ Inconclusive → Blood drawn for ELISA?.................... ☐ Yes ☐ No
   - ☐ Negative
   - ☐ Not done

   4b. Orasure test taken?... ☐ Yes ☐ No

Follow-up

5. If <5 years old, referred for assessment?................................ ☐ Yes ☐ No ☐ Not applicable

6. Referral for TB treatment needed?.. ☐ Yes ☐ No

7. Referral for HIV care needed?.. ☐ Yes ☐ No

8. Referral for ARVs needed?... ☐ Yes ☐ No

Active Case Findings - ACF Kids

Please Initial and date the appropriate section below:

1st Review:	____________ Initials	____/____/20____ Date	*Faxed by:* ____________ Initials	____/____/20____ Date
2nd Review:	____________ Initials	____/____/20____ Date	*Faxed by:* ____________ Initials	____/____/20____ Date
3rd Review:	____________ Initials	____/____/20____ Date	*Faxed by:* ____________ Initials	____/____/20____ Date
4th Review:	____________ Initials	____/____/20____ Date	*Faxed by:* ____________ Initials	____/____/20____ Date

Household ID: ☐☐☐☐ Study ID: 0 2

Child's Caregiver's Information: Lab

TB Investigation

1. Date sputum specimen collected:...................... ☐☐ (dd) ☐☐☐ (MMM) 2 0 1 ☐ (yyyy)

2. Sputum smear (AFB):.. ☐ Done ☐ Not done
(If any positive, use the highest count.)

Result: ☐ Negative/Not seen ☐ Moderate (++) ☐ Scanty (+) ☐ Numerous (+++)

3. Culture:... ☐ Done ☐ Not done

3a. Result: ZN for AAFB:.................................... ☐ Positive ☐ Negative ☐ Contaminated or lab problem

3b. Number of days to positive:........... ☐☐

3c. ID of Mycobacterium:..................... ☐ M Tuberculosis ☐ Other ☐ M Avium Complex ☐ Contaminated / Lab problem

3d. Drug sensitivity:.. ☐ Done ☐ Not Done

If resistant to Isoniazid AND Rifampacin

Isoniazid: ☐ Resistant ☐ Sensitive ☐ Not done

Rifampin: ☐ Resistant ☐ Sensitive ☐ Not done

Resistant to 2nd line drugs?..................... ☐ Yes ☐ No
(If Yes, specify which drugs below)

a. ____________________

b. ____________________

c. ____________________

4. Other biological specimen:................................ ☐ Collected ☐ Not collected
(e.g. FNA, biopsy, gastric aspirate)

4a. Specify: ____________________

4b. Date obtained:.. ☐☐ (dd) ☐☐☐ (MMM) 2 0 1 ☐ (yyyy)

4c. Result:............. ☐ TB ☐ Not TB ☐ Unknown

Active Case Findings - ACF Kids

Please Initial and date the appropriate section below:

1st Review: ____________ Initials	______/______/20______ Date	*Faxed by:* ____________ Initials	______/______/20______ Date
2nd Review: ____________ Initials	______/______/20______ Date	*Faxed by:* ____________ Initials	______/______/20______ Date
3rd Review: ____________ Initials	______/______/20______ Date	*Faxed by:* ____________ Initials	______/______/20______ Date
4th Review: ____________ Initials	______/______/20______ Date	*Faxed by:* ____________ Initials	______/______/20______ Date

Household ID [ ][ ][ ][ ] Study ID [0][2]

Child's Caregiver's Information: Final TB & HIV results

6. Did the person receive a final diagnosis of TB?.. ☐ Yes ☐ No → ***Go to Q7***
Go to Q6a (if Yes)

	dd	MMM	yyyy
6a. Date of diagnosis:......................	[ ][ ]	[ ][ ][ ]	[2][0][1][ ]
6b. Date started treatment:...............	[ ][ ]	[ ][ ][ ]	[2][0][1][ ]

(Ask patient or go to clinic)

6c. Basis for decision to start TB treatment:

☐ Smear positive ☐ CXR
☐ Sputum culture positive ☐ TB symptoms
☐ Unknown ☐ Other, specify below:

____

HIV Testing

7. 1st Rapid HIV or Orasure test result:................ ☐ Positive ☐ Negative ☐ Inconclusive ☐ Not done

8. Confirmatory Rapid Result:............................... ☐ Positive ☐ Negative ☐ Inconclusive ☐ Not done

9. Final HIV Diagnosis:... ☐ Positive ☐ Negative ☐ Unknown
Go to Q9a (if Positive)

9a. Date of diagnosis:........ [ ][ ] [ ][ ][ ] [2][0][1][ ]

9b. Most Recent CD4:.. [ ][ ][ ][ ] → 9b. Date taken:................ [ ][ ] [ ][ ][ ] [2][0][1][ ]

10. Taking ARVs at initial visit?.............................. ☐ Yes ☐ No ☐ Unknown
(If Yes, complete ARV Log)

11. Requires ARVs?... ☐ Yes ☐ No ☐ Unknown
(CD4 criteria/staging criteria)

12. Referral given for ARV initiation?...................... ☐ Yes ☐ No ☐ Unknown

Active Case Findings - ACF Kids

Please Initial and date the appropriate section below:

1st Review:	____________ Initials	____/____/20____ Date	*Faxed by:* ____________ Initials	____/____/20____ Date
2nd Review:	____________ Initials	____/____/20____ Date	*Faxed by:* ____________ Initials	____/____/20____ Date
3rd Review:	____________ Initials	____/____/20____ Date	*Faxed by:* ____________ Initials	____/____/20____ Date
4th Review:	____________ Initials	____/____/20____ Date	*Faxed by:* ____________ Initials	____/____/20____ Date
