# Supplementary material for: Integrated Source Case Investigation for Tuberculosis (TB) and HIV in the Caregivers and Household Contacts of Hospitalised Young Children Diagnosed with TB in South Africa: An Observational Study
Source: PLoS One. 2015 Sep 17;10(9):e0137518. doi: 10.1371/journal.pone.0137518 (PMC4574562; doi:10.1371/journal.pone.0137518)
Supplement: S4 File — This questionnaire was used to collect information about household contacts’ TB symptoms. (PDF) [file pone.0137518.s005.pdf]

ACF-Kids (063) Plt 5 (005) Visit 1 (010)

Household ID ☐☐☐☐ Study ID ☐☐

Use ID number from HH census

Household Demographic Info

Interview Date ☐☐ *dd* ☐☐☐ *MMM* 2 0 1 ☐ *yyyy*

This form should be completed for every consenting or assenting HH member

Instructions to interviewer:

Visit attempt:... ☐ (1, 2, 3) Was a consent form signed *(or assent if child)*...... ☐ Yes ☐ No → ***If No, STOP and do consent***

Demographic information

1. Sex:......................... ☐ Male ☐ Female

2. Date of birth:........... ☐☐☐ MMM ☐☐☐☐ yyyy **OR**, if exact date of birth not known, estimated age:.................................. ☐☐ yrs

3. Initials *(first/middle/last)*... ☐☐☐ First/Middle/Last

4. How long have you been living in this house?....................................... ☐☐ . ☐☐ yrs

5. About how many hours per day do you spend inside this house?.........a. Weekdays:....... ☐☐ hrs

b. Weekends:....... ☐☐ hrs

6. Highest grade of education attended:..... ☐☐ →

00 = No education
01-12 = Highest grade attended
13 = Any post school non degree/diploma
14 = Degree/Diploma

7. Occupational status:..
- ☐ Self-employed
- ☐ Student
- ☐ Salaried worker
- ☐ Other,specify: ________________
- ☐ Unemployed but able to work
- ☐ Unemployed and unable to work
- ☐ Occasional work (piece Jobs)

8. Sputum specimen taken from caregiver?. ☐ Yes ☐ No

8a. Did you induce a specimen?............. ☐ Yes ☐ No

8b. Specimen bar code number?............ ☐☐☐☐☐☐☐☐

8b. Date specimen taken:...................... ☐☐ dd ☐☐☐ MMM 2 0 1 ☐ yyyy

Staff Initials / Date

Active Case Findings - ACF Kids

Please Initial and date the appropriate section below:

1st Review:	____________ Initials	_____/_____/20_____ Date	*Faxed by:*	____________ Initials	_____/_____/20_____ Date
2nd Review:	____________ Initials	_____/_____/20_____ Date	*Faxed by:*	____________ Initials	_____/_____/20_____ Date
3rd Review:	____________ Initials	_____/_____/20_____ Date	*Faxed by:*	____________ Initials	_____/_____/20_____ Date
4th Review:	____________ Initials	_____/_____/20_____ Date	*Faxed by:*	____________ Initials	_____/_____/20_____ Date

Household ID ☐☐☐☐ Study ID ☐☐

Household Demographic Info

9. Do you currently have TB?.................... ☐ Yes (Go to Q9a) ☐ No ☐ Don't know (Go to Q9e)

9a. Date of diagnosis... ☐☐ dd ☐☐☐ MMM 2 0 ☐☐ yyyy

9b. Do you have a TB treatment card?... ☐ Yes ☐ No

9c. Treatment card visualised?............... ☐ Yes ☐ No

9d. Treatment status?............................ ☐ Currently on treatment ☐ Interrupted/Defaulted *Have not completed >6 months of TB treatment*

(Go to TB History and Risk.)

9e. Symptoms present at visit. *(Ask about each symptom separately.)*

	No	Yes	Duration in days
9e1. Cough................................	☐	☐ →	☐☐☐
9e2. Coughing sputum................	☐	☐ →	☐☐☐
9e3. Coughing blood..................	☐	☐ →	☐☐☐
9e4. Weight loss........................	☐	☐ →	☐☐☐
9e5. Fever.................................	☐	☐ →	☐☐☐
9e6. Soaking night sweats..........	☐	☐ →	☐☐☐
9e7. Shortness of breath.............	☐	☐ →	☐☐☐
9e8. Loss of appetite..................	☐	☐ →	☐☐☐

9f. Date of first onset of these symptoms:.................................... ☐☐ dd ☐☐☐ MMM 2 0 ☐☐ yyyy

9g. If any TB symptoms present:

9g1. Date first sought medical help for these TB symptoms:... ☐☐ dd ☐☐☐ MMM 2 0 ☐☐ yyyy

OR: Check this box if did not seek medical help:.......... ☐

9g2. Number of visits made for medical help for these symptoms:.. ☐☐

9g2a. Type of medical help sought: *(Tick all that apply)*

☐ Hospital ☐ Primary care clinic ☐ Pharmacist

☐ TB clinic ☐ VCT centre ☐ Traditional healer

☐ Other, specify: ____________________

Active Case Findings - ACF Kids

Please Initial and date the appropriate section below:

1st Review:	____________ Initials	______/______/20______ Date		*Faxed by:*	____________ Initials	______/______/20______ Date	
2nd Review:	____________ Initials	______/______/20______ Date		*Faxed by:*	____________ Initials	______/______/20______ Date	
3rd Review:	____________ Initials	______/______/20______ Date		*Faxed by:*	____________ Initials	______/______/20______ Date	
4th Review:	____________ Initials	______/______/20______ Date		*Faxed by:*	____________ Initials	______/______/20______ Date	

Household ID ☐☐☐☐ **Study ID** ☐☐

Household Demographic Info

1. Did you ever have TB before in your life?................................ ☐ Yes ☐ No

   Go to Q1a (Yes) ***Go to Q2*** (No)

   1a. What was the date of your most recent TB diagnosis?...... MMM ☐☐☐ yyyy ☐☐☐☐

   1b. Did you complete the full TB treatment ?.......................... ☐ Yes ☐ No

   1c. How many months did you take TB treatment for?........... ☐☐

2. Do you currently smoke tobacco?... ☐ Yes ☐ No

   Go to Q2a (Yes) ***Go to Q3*** (No)

   2a. How many cigarettes or pipes per day?............................ ☐☐

   2b. When did you start?.. ☐☐☐☐ year

   2c. Do you smoke inside your home?..................................... ☐ Yes ☐ No

HIV information

3. Have you previously tested **positive** for HIV?......................... ☐ Yes ☐ No

   Go to Q3a (Yes) ***Go to Household testing*** (No)

   3a. What was the date?.. dd ☐☐ MMM ☐☐☐ yyyy ☐☐☐☐

   3b. Are you currently taking ARVs?....................................... ☐ Yes ☐ No

Household testing for this person

4. Consent for VCT?... ☐ Yes ☐ No → ***Go to Q4b***

   4a. Rapid test results:
   - ☐ 2 Positive Rapids → Blood drawn for CD4 count?............. ☐ Yes ☐ No
   - ☐ Inconclusive → Blood drawn for ELISA?.................... ☐ Yes ☐ No
   - ☐ Negative
   - ☐ Not done

   4b. Orasure test taken?.. ☐ Yes ☐ No

Follow-up

5. If <5 years old, referred for assessment?................................ ☐ Yes ☐ No ☐ Not applicable

6. Referral for TB treatment needed?.. ☐ Yes ☐ No

7. Referral for HIV care needed?.. ☐ Yes ☐ No

8. Referral for ARVs needed?.. ☐ Yes ☐ No

Active Case Findings - ACF Kids

Please Initial and date the appropriate section below:

1st Review: ____________ Initials	______/______/20______ Date	*Faxed by:* ____________ Initials	______/______/20______ Date
2nd Review: ____________ Initials	______/______/20______ Date	*Faxed by:* ____________ Initials	______/______/20______ Date
3rd Review: ____________ Initials	______/______/20______ Date	*Faxed by:* ____________ Initials	______/______/20______ Date
4th Review: ____________ Initials	______/______/20______ Date	*Faxed by:* ____________ Initials	______/______/20______ Date

Household ID ☐☐☐☐ Study ID ☐☐

Household Demographic Info: Lab

TB Investigation

1. Date sputum specimen collected:...................... ☐☐ (dd) ☐☐☐ (MMM) 2 0 1 ☐ (yyyy)

2. Sputum smear (AFB):.. ☐ Done ☐ Not done
(If any positive, use the highest count.)

Result: ☐ Negative/Not seen ☐ Moderate (++) ☐ Scanty (+) ☐ Numerous (+++)

3. Culture:... ☐ Done ☐ Not done

3a. Result: ZN for AAFB:.................................... ☐ Positive ☐ Negative ☐ Contaminated or lab problem

3b. Number of days to positive:........... ☐☐

3c. ID of Mycobacterium:..................... ☐ M Tuberculosis ☐ Other ☐ M Avium Complex ☐ Contaminated / Lab problem

3d. Drug sensitivity:.. ☐ Done ☐ Not Done

Isoniazid: ☐ Resistant ☐ Sensitive ☐ Not done

Rifampin: ☐ Resistant ☐ Sensitive ☐ Not done

If resistant to Isoniazid AND Rifampacin

Resistant to 2nd line drugs?..................... ☐ Yes ☐ No
(If Yes, specify which drugs below)

a. ______________________

b. ______________________

c. ______________________

4. Other biological specimen:................................ ☐ Collected ☐ Not collected
(e.g. FNA, biopsy, gastric aspirate)

4a. Specify: ______________________

4b. Date obtained:.. ☐☐ (dd) ☐☐☐ (MMM) 2 0 1 ☐ (yyyy)

4c. Result:............. ☐ TB ☐ Not TB ☐ Unknown

Staff Initials / Date

Active Case Findings - ACF Kids

Please Initial and date the appropriate section below:

1st Review:	____________ Initials	______/______/20______ Date	*Faxed by:* ____________ Initials	______/______/20______ Date
2nd Review:	____________ Initials	______/______/20______ Date	*Faxed by:* ____________ Initials	______/______/20______ Date
3rd Review:	____________ Initials	______/______/20______ Date	*Faxed by:* ____________ Initials	______/______/20______ Date
4th Review:	____________ Initials	______/______/20______ Date	*Faxed by:* ____________ Initials	______/______/20______ Date

Household ID ☐☐☐☐ **Study ID** ☐☐

Household Demographic Info: Lab

6. Did the person receive a final diagnosis of TB?.. ☐ Yes ☐ No → ***Go to Q7***

Go to Q6a

	dd	MMM	yyyy
6a. Date of diagnosis:......................	☐☐	☐☐☐	2 0 1 ☐
6b. Date started treatment:...............	☐☐	☐☐☐	2 0 1 ☐

(Ask patient or go to clinic)

6c. Basis for decision to start TB treatment:

☐ Smear positive ☐ CXR

☐ Sputum culture positive ☐ TB symptoms

☐ Unknown ☐ Other, specify below:

____

HIV Testing

7. 1st Rapid HIV or Orasure test result:................. ☐ Positive ☐ Negative ☐ Inconclusive ☐ Not done

8. Confirmatory Rapid Result:.............................. ☐ Positive ☐ Negative ☐ Inconclusive ☐ Not done

9. Final HIV Diagnosis:... ☐ Positive ☐ Negative ☐ Unknown

→ ***Go to Q9a***

9a. Date of diagnosis:........ ☐☐ ☐☐☐ 2 0 1 ☐

9b. Most Recent CD4:.. ☐☐☐☐ → 9b. Date taken:................ ☐☐ ☐☐☐ 2 0 1 ☐

10. Taking ARVs at initial visit?.............................. ☐ Yes ☐ No ☐ Unknown
(If Yes, complete ARV Log)

11. Requires ARVs?... ☐ Yes ☐ No ☐ Unknown
(CD4 criteria/staging criteria)

12. Referral given for ARV initiation?...................... ☐ Yes ☐ No ☐ Unknown

Active Case Findings - ACF Kids

Please Initial and date the appropriate section below:

1st Review: ____________ Initials	______/______/20______ Date	*Faxed by:* ____________ Initials	______/______/20______ Date
2nd Review: ____________ Initials	______/______/20______ Date	*Faxed by:* ____________ Initials	______/______/20______ Date
3rd Review: ____________ Initials	______/______/20______ Date	*Faxed by:* ____________ Initials	______/______/20______ Date
4th Review: ____________ Initials	______/______/20______ Date	*Faxed by:* ____________ Initials	______/______/20______ Date
