# Supplementary material for: Integrated Source Case Investigation for Tuberculosis (TB) and HIV in the Caregivers and Household Contacts of Hospitalised Young Children Diagnosed with TB in South Africa: An Observational Study
Source: PLoS One. 2015 Sep 17;10(9):e0137518. doi: 10.1371/journal.pone.0137518 (PMC4574562; doi:10.1371/journal.pone.0137518)
Supplement: S5 File — This questionnaire was used to collect information about the physical structure of the household dwelling. (PDF) [file pone.0137518.s006.pdf]

ACF-Kids (063) Plt 11 (011) Visit 1 (010)

Household ID [ ][ ][ ][ ] Study ID [0][1]

Housing Information

Visit Date [ ][ ] dd [ ][ ][ ] MMM [2][0][1][ ] yyyy

This form must be completed once per household

1. What type of house is it?
   - ☐ House/Townhouse/Flat *(Family or Self owned [Brick])*
   - ☐ Hostel
   - ☐ Shack *(Informal settlement)*
   - ☐ Other, specify: ____________________
   - ☐ Shack *(Backyard)*
   - ☐ Traditional hut

2. Total number of rooms in house:.......... [ ][ ]
3. Total number of windows:................... [ ][ ]
4. Number of doors leading outside:........ [ ][ ]
5. Type of indoor heating/cooking fuel:..... [ ]
6. Electricity at home:............................ ☐ Yes ☐ No
7. Drinking water in this home:................ [ ]
8. Evidence of indoor smoking:............... ☐ Yes ☐ No
   - ☐ Person observed smoking inside during household visit
   - ☐ Smell of cigarette smoke in house during household visit
   - ☐ Visible ashtrays and/or cigarettes inside
   - ☐ Other, specify: ____________________

Fuel type codes:

1 = Gas
2 = Wood
3 = Electricity
4 = Paraffin
5 = Other

Drinking water codes:

1 = Shared tap in the street
2 = Piped water into house
3 = River or dam
4 = Share water pump in the street
5 = Other

9. Total number of people in this HH?..... [ ][ ]

HH census: *List household members living in this structure (First 4 digits same as HHID. Do not include caregiver)*
Draw a line through the UNUSED Study ID fields below.

	Study ID:		Initials *(first/last)*		Study ID:		Initials *(first/last)*
1.	[ ][ ][ ][ ]	[ ][ ] →	[ ][ ]	8.	[ ][ ][ ][ ]	[ ][ ] →	[ ][ ]
2.	[ ][ ][ ][ ]	[ ][ ] →	[ ][ ]	9.	[ ][ ][ ][ ]	[ ][ ] →	[ ][ ]
3.	[ ][ ][ ][ ]	[ ][ ] →	[ ][ ]	10.	[ ][ ][ ][ ]	[ ][ ] →	[ ][ ]
4.	[ ][ ][ ][ ]	[ ][ ] →	[ ][ ]	11.	[ ][ ][ ][ ]	[ ][ ] →	[ ][ ]
5.	[ ][ ][ ][ ]	[ ][ ] →	[ ][ ]	12.	[ ][ ][ ][ ]	[ ][ ] →	[ ][ ]
6.	[ ][ ][ ][ ]	[ ][ ] →	[ ][ ]	13.	[ ][ ][ ][ ]	[ ][ ] →	[ ][ ]
7.	[ ][ ][ ][ ]	[ ][ ] →	[ ][ ]	14.	[ ][ ][ ][ ]	[ ][ ] →	[ ][ ]

Active Case Findings - ACF Kids

Please Initial and date the appropriate section below:

1st Review:	____________ Initials	______/______/20______ Date		*Faxed by:*	____________ Initials	______/______/20______ Date	
2nd Review:	____________ Initials	______/______/20______ Date		*Faxed by:*	____________ Initials	______/______/20______ Date	
3rd Review:	____________ Initials	______/______/20______ Date		*Faxed by:*	____________ Initials	______/______/20______ Date	
4th Review:	____________ Initials	______/______/20______ Date		*Faxed by:*	____________ Initials	______/______/20______ Date	
