# Supplementary material for: Integrated Source Case Investigation for Tuberculosis (TB) and HIV in the Caregivers and Household Contacts of Hospitalised Young Children Diagnosed with TB in South Africa: An Observational Study
Source: PLoS One. 2015 Sep 17;10(9):e0137518. doi: 10.1371/journal.pone.0137518 (PMC4574562; doi:10.1371/journal.pone.0137518)
Supplement: S6 File — This paper describes the decreasing prevalence of HIV infection in young infants admitted to the Chris Hani Baragwanath Academic Hospital. (PDF) [file pone.0137518.s007.pdf]

The prevalence of malnutrition in children admitted to a general paediatric ward at the Chris Hani Baragwanath Academic Hospital: A cross-sectional survey

J Brink, MB ChB; **J M Pettifor**, MB BCh, PhD(Med), FCPaed(SA);
S G Lala, MB BCh, DCH(SA), DTM&H, FCPaed (SA), MMed, PhD

Department of Paediatrics, School of Clinical Medicine, Faculty of Health Sciences, University of the Witwatersrand and Chris Hani Baragwanath Academic Hospital, Soweto, Johannesburg, South Africa

Corresponding author: *S G Lala (sanjay.lala@wits.ac.za)*

Background. The prevalence of malnutrition, an important contributor to childhood mortality, is poorly described in hospitalised South African (SA) children, many of whom are HIV-exposed or HIV-infected.
Objectives. To describe the prevalence of malnutrition in infants and children <14 years of age admitted to a general paediatric ward at the Chris Hani Baragwanath Academic Hospital in Soweto, SA, and to compare the nutritional status of infants <18 months of age who were HIV-unexposed, HIV-exposed but uninfected (HEU) or HIV-infected.
Methods. A cross-sectional nutritional survey was conducted on 222 admitted children. A total of 139 infants were <18 months of age.
Results. Stunting was the most common form of malnutrition (40.5%), followed by underweight-for-age (33.3%) and wasting (23.4%). Of 175 children aged <5 years, 22 (12.6%) were severely wasted. Twenty-four (10.8%) children were HIV-infected: 6 children were <18 months, 3 were ≥18 months but <5 years and 15 children were ≥5 years. For children ≤18 months, HEU children (n=56) were significantly more underweight and stunted than their HIV-unexposed peers (n=77); weight-for-age and height-for-age median z-scores for these groups were −1.81 v. −0.63 (p=0.0038) and −2.51 v. −0.51 (p=0.004), respectively.
Conclusion. Malnutrition is prevalent in hospitalised children, with stunting being the most common form. The prevalence of HIV-infection is decreasing in younger children, but HEU children, who constitute a large proportion of total hospital admissions, have high rates of malnutrition, especially stunting.

S Afr J CH 2014;8(3):112-116. DOI:10.7196/SAJCH.787.

Length (or height) and weight are anthropometric parameters that should be routinely measured on children admitted to general paediatric wards in South Africa (SA). These measurements assist in the identification of childhood malnutrition, which has been described as 'the most important risk factor for illness and death in young children'.[1] Malnutrition is usually detected as a comorbid condition in admitted children, although some are admitted with a primary diagnosis of malnutrition. The identification and accurate categorisation of malnourished children, who are likely to have longer hospital stays than adequately nourished children and suffer from high mortality rates,[1] are therefore of utmost importance to the clinician because specific therapeutic regimens are used to treat malnourished children.[2]

There is a paucity of data defining the prevalence and specific types of malnutrition in hospitalised children in SA.[3] It was expected that a large proportion of the admitted children would be stunted because this is the most common form of malnutrition in SA.[4] Further, some wasted children would have been specifically admitted for the treatment of severe acute malnutrition (SAM). Nonetheless, it is useful to define the prevalence and identify the specific types of malnutrition in hospitalised children because anthropological assessments may provide information on possible diagnosis and help to identify specific groups of children who are at risk from increased morbidity or mortality. Hospital-based anthropometric surveys are a convenient way to monitor changing patterns (but not prevalence) of malnutrition in the community.

HIV-infected children, who have over the last 3 decades constituted a major proportion of total admissions,[5-8] are often malnourished. It is our impression, however, that the proportion of HIV-infected children, as a percentage of the total ward admissions, is decreasing. This decrease is probably due to the improved implementation of prevention-of-mother-to-child-transmission (PMTCT) programmes. Our anecdotal experience suggests that the number of HIV-exposed-but-uninfected (HEU) children who are admitted to hospital is increasing and now comprises a substantial proportion of all paediatric admissions. The nutritional status of these children is not well defined: data from developed countries suggest that HEU children grow normally[9] but reports from sub-Saharan Africa suggest that HEU children are more malnourished than their HIV-unexposed peers.[10] Our clinical impression is that a greater proportion of HEU children are malnourished compared with HIV-unexposed children.

Accurate anthropometric measurements are required to identify stunting and wasting in children, yet our experience is that routine length/height measurements, in particular, are imprecisely done. These inaccurate measurements may affect the classification of malnutrition in hospitalised children and the consequent calculation of rates of wasting and stunting.

We therefore performed a cross-sectional anthropometric survey of children admitted to a general paediatric ward at Chris Hani Baragwanath Academic Hospital (CHBAH). We documented the HIV status of all admitted children and assessed the anthropometric status of HIV-unexposed, HIV-infected and HEU children to confirm our

hypothesis that HEU children are significantly more malnourished that their HIV-unexposed peers. We also remeasured each admitted child's length/height and compared our measurements with those made by the clinical staff. We then determined the potential effect of inaccurate measurements made by the clinical staff on the rates of wasting and stunting.

Methods

Patients

A cross-sectional survey was performed using data obtained from all children <14 years of age admitted to a general medical paediatric ward (Ward 17) at the CHBAH during the months of May and August 2012. This ward is similar to and representative of the other three general wards, with each ward on admission duty (i.e. intake) every 4th day. Neonates and children <14 years with non-surgical conditions are admitted to the general paediatric ward, after being triaged by the paediatric emergency outpatient department and the registrars on intake. All children admitted to Ward 17 were eligible for inclusion into the study with the exception of prematurely born neonates (born before 37 completed gestational weeks) because there are no suitable growth standards for these children. This ward was chosen because the registrar routinely remeasures the child's length/height using a length board or stadiometer. The child's weight and length/height are initially measured in the admission ward by the clinical staff. The months of May (autumn) and August (late winter) were chosen partly because of convenience and also because, in Gauteng, the largest numbers of children are admitted during the annual respiratory syncytial virus epidemic, which begins in late February and lasts until the end of August.[11] The relevant data were abstracted in an anonymised manner onto a data collection form and transferred to a computerised database (a spreadsheet using Microsoft Excel) for statistical analysis. Ethical clearance was obtained from the Human Research Ethics Committee of the University of the Witwatersrand (clearance number M120958).

Measurements

The children's weights were routinely measured on an electronic scale (Seca 336 infant scale for infants and young children, and Seca 255 measuring device for older children; Seca, SA). The children's heights were measured on a calibrated Harpenden stadiometer (for children >2 years of age) or Harpenden infantometer (for children <2 years of age) (Holtain Limited, UK). For children unable to stand, a recumbent length was measured on the infantometer if length was <940 mm. If the length was ≥940 mm, the child's height was measured on the stadiometer with the help of an assistant. For length measurements using the infantometer, the head was positioned against a vertical board at one end, in the Frankfurt plane. The body of the supine infant was held extended and a second board was placed against the vertically aligned feet. For height measurements, children stood barefoot (with feet together) with their backs against the stadiometer, legs fully extended, and their heads in the Frankfurt plane. Two measurements were made, and the average was recorded.

Study definitions[12]

- **Underweight:** a child whose weight-for-age *z*-score (WAZ) is below –2 standard deviations (SDs) of the median World Health Organization (WHO) standard value.
- **Severely underweight:** a child whose WAZ is below –3 SDs of the median WHO standard value.
- **Wasted:** a child whose weight-for-length/height *z*-score (WHZ) is below –2 SDs of the median WHO standard value.
- **Severely wasted:** a child whose WHZ is below –3 SDs of the median WHO standard value.
- **Stunted:** a child whose length/height-for-age *z*-score (HAZ) is below –2 SDs of the median WHO standard value.
- **Severely stunted:** a child whose HAZ is below –3 SDs of the median WHO standard value.
- **HIV-infected:** in children >18 months of age, HIV infection was defined by a positive HIV enzyme-linked immunosorbent assay (ELISA) and/or HIV DNA polymerase chain reaction (PCR) test. In children ≤18 months, HIV infection was confirmed by a positive HIV DNA PCR test.
- **HIV-exposed:** a child was considered to be HIV-exposed if his/her mother had a positive HIV ELISA test before or during the pregnancy of that particular child.
- **HIV-uninfected:** a child was considered HIV-uninfected if his/her HIV tests were negative (negative HIV DNA PCR in children ≤18 months old; negative HIV ELISA or HIV DNA PCR in children >18 months old) together with the confirmed knowledge that his/her mother is HIV-uninfected.

Data analysis and statistics

Z-scores for weight-for-age, weight-for-length/height and length/height-for-age were calculated using the WHO Anthro and AnthroPlus software programmes (Department of Nutrition, World Health Organisation, Geneva). The prevalence of underweight, wasting and stunting was calculated as a simple rate or percentage. The median and 25 - 75% interquartile range (IQR) for each individual anthropometric parameter (e.g., stunting) was calculated using a commercial statistical programme. Median *z*-scores for wasting and stunting were compared between groups using the Kruskal-Wallis and Mann-Whitney tests. Proportions were compared using Fisher's exact test. The agreement between the length or height measurements made by the admission staff (i.e. nurses, interns and medical officers) with those made by the paediatric registrar was determined using the Bland-Altman plot. Statistical analyses were performed using GraphPad Prism version 5.00 for Windows (GraphPad Software, San Diego California, USA).

Results

A total of 264 children were admitted during the months of May and August 2012: 149 children were admitted during May and 115 during August. Twenty-four children were excluded from the analysis: 20 children were discharged before the registrar could remeasure their length/height, and 4 children, of whom 2 suffered from oedematous malnutrition, died shortly after admission. The final anthropometric analysis is reported for 222 of the remaining 240 children because 18 prematurely born neonates (all born before 37 completed gestational weeks) were excluded from the data analysis. Of the 222 children included in the final analysis, 123 (55.4%) children were male.

Ages of admitted children

The ages of admitted children ranged from 1 day to 14 years, but most children were young: the median age of admitted children was 327 days or 10.9 months (IQR 25 - 75%, 2.4 - 49.6 months). The majority of admitted children were infants (n=112; 50.4%), followed by toddlers <2 years of age (n=34; 15.3%). Children ≥2 years (n=76) made up the remaining 34.2% of admissions. In the 2 - 5 year-group, there were 29 patients (13.1%), and there were 27 patients (12.2%) in the 5 - 10-year group. Twenty children (9.0%) were ≥10 years.

General anthropometric data

In general, the admitted children were shorter and lighter than the standard median. The HAZs, WAZs and WHZs were between 0 and –2 SDs. Stunting was the most common form of malnutrition (40.5%), followed by underweight-for-age (33.3%) and wasting (23.4%) (Table 1). The prevalence of malnutrition was greatest in children >10 years old, although these children constituted a small proportion of the overall admissions.

Table 1. Anthropometric data of 222 children admitted to a general paediatric ward at the Chris Hani Baragwanath Academic Hospital

	All children	0 - 1 years	>1 - 5 years	>5 - 10 years	>10 - <14 years
No. of children	222	112	63	27	20
No. of HIV-infected children	24	2	7	6	9
Nutritional status, *n* (%)					
Normal anthropometry	95 (42.7)	51 (45.5)	24 (38.1)	16 (59.2)	4 (20.0)
Underweight	74 (33.3)	35 (31.3)	19 (30.2)	9 (33.3)	11 (55.0)
Severely underweight	41 (18.5)	18 (16.1)	13 (20.6)	5 (18.5)	5 (25.0)
Stunted	90 (40.5)	44 (39.3)	27 (42.8)	7 (25.9)	12 (60.0)
Severely stunted	46 (20.7)	23 (20.5)	14 (22.2)	4 (14.8)	5 (25.0)
Wasted	52 (23.4)	29 (25.9)	14 (22.2)	4 (14.8)	5 (25.0)
Severely wasted	28 (12.6)	14 (12.5)	8 (12.6)	3 (11.1)	3 (15.0)
Anthropometric median *z*-scores (IQR 25 - 75%)*					
WAZ	−1.23 (−2.36 - 0.05)	−1.05 (−2.09 - 0.39)	−1.31 (−2.39 - 0.06)	−1.09 (−2.35 - −0.46)	−2.14 (−3.12 - −1.17)
HAZ	−1.43 (−2.75 - 0.05)	−1.10 (−2.76 - 0.28)	−1.65 (−2.94 - 0.59)	−0.81 (−2.54 - 0.17)	−2.51 (−3.02 - −1.00)
WHZ	−0.46 (−1.87 - 0.87)	−0.21 (−2.10 - 1.17)	−0.50 (−1.89 - 1.04)	−1.30 (−1.91 - 0.10)	−1.0 (−2.15 - 0.38)

IQR = interquartile range; WAZ = weight-for-age *z*-score; HAZ = length/height-for-age *z*-score; WHZ = weight-for-length/height *z*-score.

*The median *z*-scores, assessed using the Kruskal-Wallis test, were not significantly different between the various age groups (WAZ p=0.2728; HAZ p=0.3097; WHZ p=0.2440). There were no overweight or obese children admitted during the time of the survey.

Table 2. The anthropometric data of 139 children ≤18 months of age stratified according their HIV status

	All HIV-negative children	HIV-unexposed	HIV-exposed but uninfected (HEU)	HIV-infected
No. of patients	133	77	56	6
Nutritional status, *n* (%)				
Normal anthropometry	77 (57.9)	63 (81.8)	14 (25.0)	2 (33.3)
Underweight	48 (36.1)	16 (20.8)	32 (57.1)	2 (33.3)
Severely underweight	23 (17.3)	10 (12.9)	13 (23.2)	2 (33.3)
Stunted	69 (51.8)	34 (44.2)	35 (62.5)	4 (66.7)
Severely stunted	29 (21.8)	9 (11.7)	20 (35.7)	3 (50.0)
Wasted	35 (26.3)	21 (27.3)	14 (25.0)	2 (33.3)
Severely wasted	18 (13.5)	13 (16.8)	5 (8.9)	2 (33.3)
Anthropometric median *z*-scores (IQR 25 - 75%)*				
WAZ	−1.09 (−2.14 - 0.40)	−0.63† (−1.80 - 0.60)	−1.81† (−2.70 - −0.24)	−1.74 (−5.07 - −0.72)
HAZ	−1.10 (−2.75 - 0.31)	−0.51‡ (−1.82 - 0.59)	−2.51‡ (3.60 - −0.22)	−2.42 (−4.46 - −1.14)
WHZ	−0.30 (−2.10 - 1.24)	−0.37 (−1.85 - 1.24)	−0.77 (−2.30 - 1.05)	−1.33 (−3.38 - 0.82)

IQR = interquartile range; WAZ = weight-for-age *z*-score; HAZ = length/height-for-age *z*-score; WHZ = weight-for-length/height *z*-score.

*Compared with their HIV-unexposed peers, HEU children were significantly more underweight and stunted.

† p=0.0038 (Mann-Whitney test).

‡ p=0.004 (Mann-Whitney test).

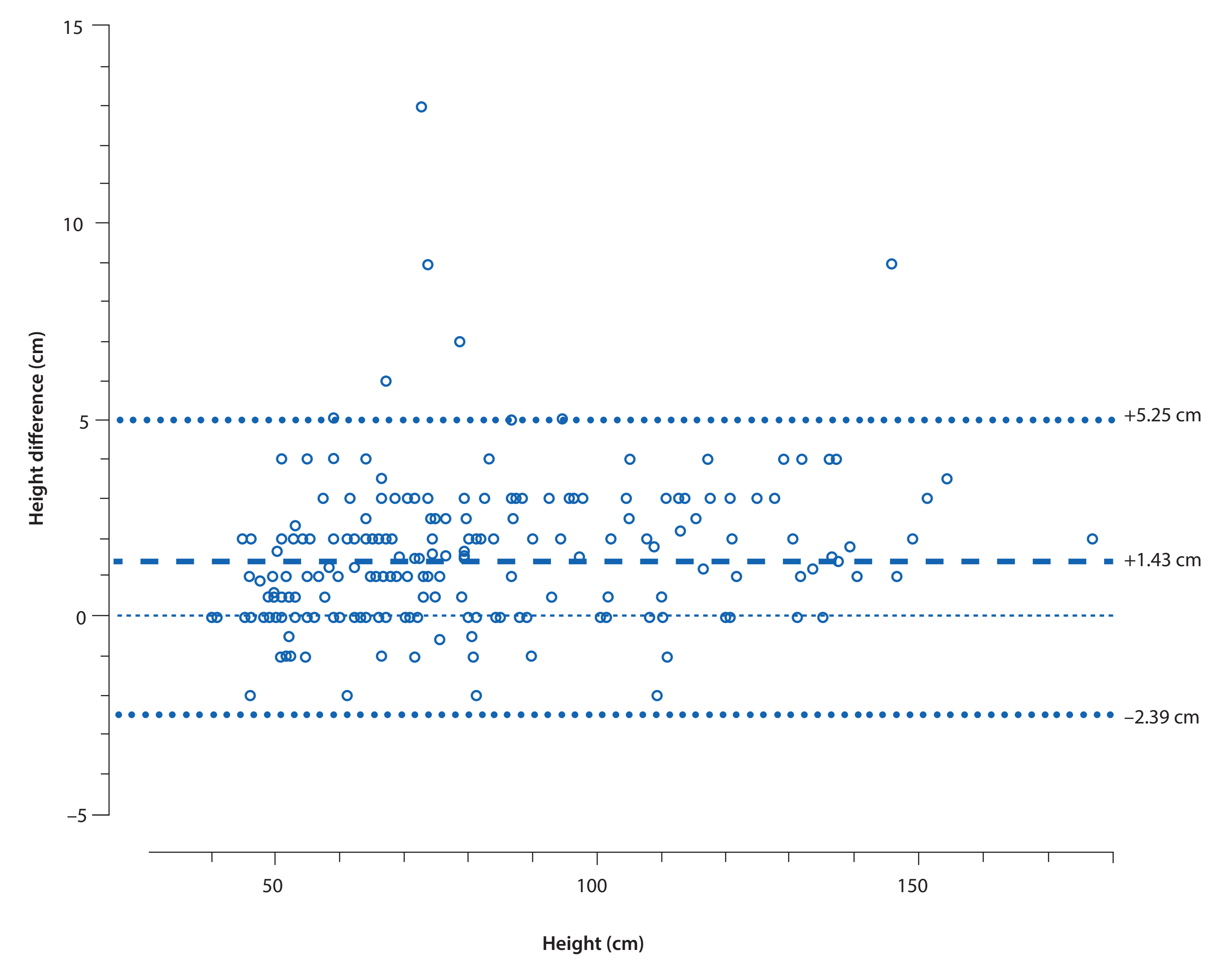

Fig. 1. Bland-Altman plot demonstrating the mean difference between the clinical record measurement and the researcher measurement (heavy dashed line). Height was the average of the clinical record ('standard') measurement and the researcher ('gold standard') measurement. The limits of agreement are also shown (heavy dotted lines), defined as the mean difference ±1.96 times the standard deviation of the difference.

Of the 175 children aged ≤5 years, 22 (12.6%) were severely wasted and therefore, by definition, suffering from SAM. Eight children (4.6%), of whom two were also severely wasted, suffered from oedematous malnutrition. About 43% of all admitted children had normal anthropometric parameters. No overweight or obese children were admitted during the study periods.

HIV prevalence

Twenty-four (10.8%) of the 222 admitted children were HIV-infected (Table 1). The proportion of HIV-infected children varied according to age: in general, HIV infection was much more prevalent in the older children compared with the younger children. The majority of HIV-infected children (n=15) were >5 years old; only two infants (1.8%) were HIV-infected.

HIV-infected children had lower anthropometric z-scores than HIV-negative children. Of the 24 HIV-infected children, only four (16.7%) had normal anthropometric parameters compared with 81 (36.5%) of the 198 HIV-negative children. The proportion of abnormal anthropometrical measurements was significantly higher in HIV-infected compared with HIV-uninfected children (p=0.0252, Fisher's exact test; odds ratio (OR) 3.4; 95% confidence interval (CI) 1.14 - 10.51).

Of the 139 children ≤18 months old, 6 were HIV-infected and 133 were HIV-negative. We further categorised HIV-negative children as either HIV-unexposed children (n=77) or HEU children (n=56). The majority of HIV-unexposed children (n=63, 81.8%) had normal anthropometric parameters compared with 14 (25.0%) of HEU children. The proportion of abnormal anthropometric measurements was significantly higher in HEU children compared with HIV-unexposed children (p<0.0001, Fisher's exact test; OR 13.50; 95% CI 5.84 - 31.20). HEU children had significantly lower HAZs and WAZs, i.e. they were significantly more underweight and stunted, compared with their HIV-unexposed peers.

Accuracy of routine length/height measurements

Clinical staff generally tended to over-estimate the length/height measurements of admitted children when compared with the measurements made by the paediatric registrar (Fig. 1). The proportion of children categorised as either stunted or wasted changed after the children's length/height was remeasured. Based on the clinical staff's measurements, 71 (31.9%) of children were classified as stunted and 66 (29.7%) as wasted. After remeasurement of length/height by the registrar, the proportion of stunted children increased to 43.6% (n=97) while the proportion of wasted children decreased

to 24.7% (n=55). The difference between routine and registrar measurements increased the prevalence of stunting by 36.7%. Of the 222 children, the average difference of height/length measurements between the clinical staff and the registrar was +1.43 cm with the 95% limits of agreement between –2.39 cm and +5.25 cm (Fig. 1).

Discussion

The results of our cross-sectional anthropometric survey indicate that malnutrition is prevalent in hospitalised children. The most common form of malnutrition was stunting, followed by underweight-for-age and wasting. The anthropometric status of non-hospitalised children <14 years of age has recently been determined by the SA National Health and Nutrition Examination Survey (SANHANES-1),[4] and our results broadly reflected these findings: in undernourished children, stunting is more prevalent than underweight-for-age and wasting. The SANHANES-1 survey found that stunting was especially prevalent in children <3 years of age (26.95% and 25.9% for boys and girls, respectively).

Twenty-eight of all admitted children were severely wasted, of whom 22 (9.9% of all admissions) were <5 years of age and thus required treatment for SAM. During our relatively short study period, no overweight or obese children were admitted, although obesity rates of up to 13% have been documented in a previous SA hospital-based study.[3] Our results are comparable with studies done in Africa[12] but, in developed countries, much lower rates of malnutrition (6.1 - 14%) are reported for hospitalised children, the majority of whom suffer from an underlying chronic illness.[13]

The prevalence of HIV infection in hospitalised children was 10.8%, which is the lowest prevalence noted at CHBAH in over 2 decades.[5-8] A declining prevalence of HIV infection in children admitted to our hospital has been previously reported; however, it appears that the rate continues to decline, because the current prevalence rate is lower than the 19% recorded in 2011.[8] Further, the prevalence of HIV infection is higher in older children compared with younger children; this difference is most likely due to the success of the PMTCT programmes in SA. The nutritional status of HIV-infected children was worse than the nutritional status of HIV-uninfected children, but the small numbers of HIV-infected children make it difficult to determine whether this difference was significant.

We had hypothesised that the nutritional status of HEU children would be worse than that of HIV-unexposed children, and our findings support this hypothesis. The nutritional status of HEU children was closer to that of HIV-infected children than of HIV-unexposed children. We chose to include only children <18 months of age for this analysis because we assumed that the present maternal HIV status would most likely reflect the maternal HIV status at the time of delivery. We are uncertain of the reasons for the poor nutritional status of HEU children but we speculate that these children may be exposed to more infectious pathogens via their infected mothers or are more likely to live in poorer socio-economic conditions. Our findings are consistent with studies reporting that the nutritional status of African HEU children is worse than HIV-unexposed children,[10] in contrast to European HEU children, who maintain good nutrition.[9]

The importance of measuring length accurately is underscored by our findings. Wasting and stunting are potentially misclassified by inaccurate measurements, and this has therapeutic implications, for example, in the identification of those malnourished children who should be treated for SAM. Our study is not unique in having found poor accuracy of height/length measurements as part of the routine assessment of children on admission,[14] but it does help to emphasise the need for accuracy in busy acute paediatric wards, where the measurements are often assigned to the most junior and least trained of the clinical ward staff, and stadiometers are often broken or not available.

Children with lower respiratory tract infections and gastroenteritis accounted for the majority (51%) of all admissions, but we did not detect any associations between the rates of malnutrition and these diseases. We did not detect any significant association between the specific types of malnutrition and the length of hospitalisation, although those children who stayed for over 20 days were all stunted.

Study limitations

The limitations of our study include the relatively small number of measurements performed over a brief period of time, although our results are probably representative of the nutritional status of children admitted to CHBAH. As height was not properly measured, it is possible that weight was also not properly assessed and we had no way to ascertain that the weighing scales were calibrated and serviced regularly. Nonetheless, our findings emphasise the importance of accurate measurements to identify and specify the type of malnutrition in hospitalised children.

Conclusion

Our study shows that malnutrition, especially stunting, is prevalent in hospitalised children. It is encouraging to see the continuing fall in prevalence of HIV infection among admitted children, but the poor nutritional status of HEU children is worrying: these children constitute a major proportion of all admissions and are at risk for complications related to malnutrition. Our findings need to be verified in other SA settings to determine whether HEU children should be considered as an at-risk group who should receive nutritional intervention early on in life.

Acknowledgements. S G Lala was funded by a Faculty Research Committee Individual Grant from the Faculty of Health Sciences of the University of the Witwatersrand.

References

1. Müller O, Krawinkel M. Malnutrition and health in developing countries. CMAJ 2005;173(3):279-286. [http://dx.doi.org/10.1503/cmaj.050342]
2. Duggan MB. Anthropometry as a tool for measuring malnutrition: Impact of the new WHO growth standards and reference. Ann Trop Paediatr 2010;30(1):1-17. [http://dx.doi.org/10.1179/146532810X12637745451834]
3. Marino LV, Goddard E, Workman L. Determining the prevalence of malnutrition in hospitalised paediatric patients. S Afr Med J 2006;96(9 Pt 2):993-995.
4. Shisana O, Labadarios D, Rehle T, et al. South African National Health and Nutrition Examination Survey (SANHANES-1). Cape Town: HSRC Press, 2013.
5. Zwi KJ, Pettifor JM, Soderlund N. Paediatric hospital admissions at a South African urban regional hospital: The impact of HIV, 1992-1997. Ann Trop Paediatr 1999;19(2):135-142.
6. Zwi KJ, Pettifor JM, Soderlund N, et al. HIV infection and in-hospital mortality at an academic hospital in South Africa. Arch Dis Child 2000;83(3):227-230.
7. Meyers TM, Pettifor JM, Gray GE, et al. Pediatric admissions with human immunodeficiency virus infection at a regional hospital in Soweto, South Africa. J Trop Pediatr 2000;46(4):224-230.
8. Meyers T, Dramowski A, Schneider H, et al. Changes in pediatric HIV-related hospital admissions and mortality in Soweto, South Africa, 1996-2011: Light at the end of the tunnel? J Acquir Immune Defic Syndr 2012;60(5):503-510. [http://dx.doi.org/10.1097/QAI.0b013e318256b4f8]
9. Newell ML, Borja MC, Peckham C, et al. Height, weight, and growth in children born to mothers with HIV-1 infection in Europe. Pediatrics 2003;111(1):e52-60. [http://dx.doi.org/10.1542/peds.111.1.e52]
10. Filteau S. The HIV-exposed, uninfected African child. Trop Med Int Health 2009;14(3):276-287. [http://dx.doi.org/10.1111/j.1365-3156.2009.02220.x]
11. Green RJ, Zar HJ, Jeena PM, Madhi SA, Lewis H. South African guideline for the diagnosis, management and prevention of acute viral bronchiolitis in children. S Afr Med J 2010;100(5):320,322-325.
12. De Onis M, Blössner M. The World Health Organization Global Database on Child Growth and Malnutrition: Methodology and applications. Int J Epidemiol 2003;32(4):518-526.
13. Joosten KF, Hulst JM. Malnutrition in pediatric hospital patients: Current issues. Nutrition 2011;27(2):133-137. [http://dx.doi.org/10.1016/j.nut.2010.06.001]
14. Howe LD, Tilling K, Lawlor DA. Accuracy of height and weight data from child health records. Arch Dis Child 2009;94(12):950-954. [http://dx.doi.org/10.1136/adc.2009.162552]
