# Supplementary material for: Integrated Source Case Investigation for Tuberculosis (TB) and HIV in the Caregivers and Household Contacts of Hospitalised Young Children Diagnosed with TB in South Africa: An Observational Study
Source: PLoS One. 2015 Sep 17;10(9):e0137518. doi: 10.1371/journal.pone.0137518 (PMC4574562; doi:10.1371/journal.pone.0137518)
Supplement: S1 Table — (DOCX) [file pone.0137518.s008.docx]

**Results**

**Predictors of newly‑diagnosed TB disease in caregivers and household contacts (S1-S3 Tables)**

Among caregivers, univariate analysis suggested that the following variables increased the likelihood of detecting newly‑diagnosed TB: (i) TB symptoms (OR 4·33; 95% CI: 1·85 to 10·11); (ii) other HIV-infected individuals living in the household (OR 1·65; 95% CI: 1·07 to 2·53); and (iii) other household members with TB symptoms (OR 1·63; 95% CI: 1·24 to 2·14). After adjustment by multivariate analysis, the presence of TB symptoms remained a significant risk factor for detecting newly‑diagnosed TB (adjusted OR: 5·07; 95% CI: 2·09 to 12·33). While HIV infection was associated with increased odds of new TB infection, this association was not statistically significant after adjustment by multivariate analysis (adjusted OR: 2·89; 95% CI: 0·91 to 9·11). Laboratory‑confirmed TB in the sentinel paediatric case was not associated with newly‑diagnosed TB disease in the caregiver.

Among non-caregiver household contacts, univariate analysis suggested that the following variables increased the likelihood of detecting newly‑diagnosed TB: (i) the number of household contacts with TB symptoms (OR: 1·47; 95% CI: 1·19 to 1·82); (ii) monthly household income (OR: 0·76; 95% CI: 0·58 to 0·99); and (iii) the number of individuals per window or door in the household (OR: 1·29; 95% CI: 1·05 to 1·59). After adjustment by multivariate analysis, household conditions predisposing to air-borne transmission of pathogens (i.e. the number of individuals per window or door) remained a significant risk factor for detecting laboratory confirmed TB in household contacts (adjusted OR: 1·29; 95% CI: 1·05 to 1·59).

S1 Table. Predictors of newly‑diagnosed TB disease among caregivers of children with TB.

| **Variable** | **Unadjusted Odds Ratio (95% CI)** | **Adjusted Odds Ratio (95% CI)** |
| --- | --- | --- |
| Age, Sentinel Case (Months) | 1·00 (0·97 to 1·02) | ---- |
| Age, Caregiver (Years) | 1·02 (0·97 to 1·07) | 0·99 (0·92 to 1·07) |
| Male gender | 3·20 (0·69 to 14·81) | ---- |
| Less than 8th Grade Education | 1·12 (0·37 to 3·37) | 0·81 (0·24 to 2·68) |
| Unemployed | 1·18 (0·45 to 3·06) | 1·10 (0·45 to 2·68) |
| Smoker | 0·58 (0·08 to 4·43) | 0·50 (0·04 to 5·87) |
| Average hours in the house per week | 0·99 (0·98 to 1·00) | ---- |
| Lab Confirmation, Sentinel Case | 0·72 (0·16 to 3·12) | 0·91 (0·20 to 4·08) |
| TB Symptoms present in Caregiver | 4·33 (1·85 to 10·11) | **5**·**07 (2**·**09 to 12**·**33)** |
| Previous TB in Caregiver | 2·63 (0·85 to 8·09) | 0·83 (0·19 to 3·72) |
| HIV Infection in Caregiver | 2·15 (0·79 to 5·89) | 2·89 (0·91 to9·11) |
| Reported a HH member with cough | 1·39 (0·58 to 3·34) | ---- |
| HH Income (per 1000 Rands) | 1·02 (0·96 to 1·07) | 1·25 (1·00 to 1·58) |
| Number of HH contacts | 0·99 (0·82 to 1·21) | 0·97 (0·77 to 1·21) |
| Number of HH contacts with HIV | 1·65 (1·07 to 2·53)* | ---- |
| At least 1 HH contact ≥65 years old | 2·03 (0·86 to 4·80) | 3·99 (0·99 to 16·14) |
| Number of HH contacts with TB symptoms | 1·63 (1·24 to 2·14)* | ---- |
| Live in House/Townhouse | 1·27 (0·55 to 2·94) | ---- |
| Persons per Room | 0·67 (0·47 to 0·95)* | ---- |
| Persons per Window/Door | 0·68 (0·41 to 1·12) | ---- |

HH= household

*Some variables that were significant on univariate analysis were excluded in the final model because they: (i) were not significant in the final model; (ii) contained substantial missing data (>10% of observations missing); and (iii) were collinear with other variables included in the model.
