# Supplementary material for: Integrated Source Case Investigation for Tuberculosis (TB) and HIV in the Caregivers and Household Contacts of Hospitalised Young Children Diagnosed with TB in South Africa: An Observational Study
Source: PLoS One. 2015 Sep 17;10(9):e0137518. doi: 10.1371/journal.pone.0137518 (PMC4574562; doi:10.1371/journal.pone.0137518)
Supplement: S2 Table — (DOCX) [file pone.0137518.s009.docx]

S2 Table. Predictors of newly‑diagnosed TB disease among non-caregiver household contacts of children with TB.

| **Variable** | **Unadjusted Odds Ratio (95% CI)** | **Adjusted Odds Ratio (95% CI)** |
| --- | --- | --- |
| Age, Sentinel Case (Months) | 1·00 (0·98 to 1·01) | ---- |
| Age, Caregiver (Years) | 1·00 (0·99 to 1·02) | 1·01 |
| Male gender | 0·89 (0·47 to 1·68) | 0·60 (0·24 to 1·51) |
| Less than 8th Grade Education | 1·55 (0·78 to 3·09) | 1·22 (0·51 to 2·91) |
| Unemployed | 0·81 (0·32 to 2·06) | 0·43 (0·16 to 1·15) |
| Smoker | 1·40 (0·64 to 3·07) | 1·77 (0·75 to 4·19) |
| Average hours in the house per week | 1·19 (0·64 to 2·21) | ---- |
| Lab Confirmation, Sentinel Case | 1·61 (0·74 to 3·53) | 1·66 (0·58 to 4·70) |
| TB Symptoms present in Caregiver | 1·54 (0·71 to 3·37) | 1·17 (0·41 to 3·36) |
| Previous TB in Caregiver | 2·42 (0·88 to 6·62) | 1·13 (0·35 to 3·67) |
| HIV Infection in Caregiver | 2·27 (1·18 to 4·36) | **2**·**80 (1**·**21 to 6**·**46)** |
| Reported a HH member with cough | 1·80 (0·84 to 3·83) | ---- |
| HH Income (per 1000 Rands) | 0·76 (0·58 to 0·99)* | 0·64 (0·40 to 1·05) |
| Number of HH contacts | 1·07 (0·92 to 1·24) | ---- |
| Number of HH contacts with HIV | 1·70 (1·11 to 2·59)* | ---- |
| At least 1 HH contact ≥65 years old | 0·85 (0·31 to 2·33) | 1·45 (0·49 to 4·30) |
| Number of HH contacts with TB symptoms | 1·47 (1·19 to 1·82)* | ---- |
| Live in House/Townhouse | 1·30 (0·63 to 2·69) | ---- |
| Persons per Room | 1·20 (0·99 to 1·45) | ---- |
| Persons per Window/Door | 1·29 (1·05 to 1·59) | **1**·**35 (1**·**04 to 1**·**75)** |

HH= household

*Some variables that were significant on univariate analysis were excluded in the final model because they: (i) were not significant in the final model; (ii) contained substantial missing data (>10% of observations missing); and (iii) were collinear with other variables included in the model.
