# Supplementary material for: Integrated Source Case Investigation for Tuberculosis (TB) and HIV in the Caregivers and Household Contacts of Hospitalised Young Children Diagnosed with TB in South Africa: An Observational Study
Source: PLoS One. 2015 Sep 17;10(9):e0137518. doi: 10.1371/journal.pone.0137518 (PMC4574562; doi:10.1371/journal.pone.0137518)
Supplement: S3 Table — (DOCX) [file pone.0137518.s010.docx]

S3 Table. Predictors of newly‑diagnosed TB disease among all contacts (caregivers and non-caregiver household members)

| **Variable** | **Unadjusted Odds Ratio (95% CI)** | **Adjusted Odds Ratio (95% CI)** |
| --- | --- | --- |
| Age, Sentinel Case (Months) | 1·00 (0·98 to 1·01) | ---- |
| Age, All contacts (Years) | 1·01 (0·99 to 1·02) | 1·00 (0·98 to 1·02) |
| Male gender | 1·13 (0·67 to 1·91) | 1·25 (0·73 to 2·14) |
| Less than 8th Grade Education | 1·23 (0·72 to 2·11) | 1·35 (0·74 to 2·44) |
| Unemployed | 0·98 (0·57 to 1·69) | 0·66 (0·36 to 1·21) |
| Smoker | 1·15 (0·56 to 2·38) | 1·03 (0·49 to 2·15) |
| Average hours in the house per week | 0·99 (0·98 to 1·00) | ---- |
| Lab Confirmation, Sentinel Case | 1·28 (0·65 to 2·54) | 1·28 (0·63 to 2·58) |
| TB Symptoms present in any contact | 2·25 (1·31 to 3·86) | **2**·**01 (1**·**11 to 3**·**62)** |
| Previous TB in any contact | 2·55 (1·16 to 5·62) | 1·56 (0·64 to 3·78) |
| HIV Infection in any contact | 2·07 (1·27 to 3·38) | **2**·**16 (1**·**20 to 3**·**90)** |
| Any contact reported with cough | 1·62 (0·85 to 3·07) | ---- |
| HH Income (per 1000 ZAR) | 0·93 (0·76 to 1·13) | 0·94 (0·75 to 1·16) |
| Number of HH contacts | 1·03 (0·91 to 1·16) | ---- |
| Number of HH contacts with HIV | 1·64 (1·12 to 2·40)* | ---- |
| At least 1 HH contact ≥65 years old | 1·16 (0·50 to 2·70) | 1·25 (0·47 to 3·34) |
| Number of HH contacts with TB symptoms | 1·48 (1·23 to 1·78)* | ---- |
| Live in House/Townhouse | 1·25 (0·69 to 2·27) | ---- |
| Persons per Room | 1·05 (0·86 to 1·28) | ---- |
| Persons per Window/Door | 1·12 (0·88 to 1·42) | 1·10 (0·88 to 1·36) |

HH= household

*Some variables that were significant on univariate analysis were excluded in the final model because they: (i) were not significant in the final model; (ii) contained substantial missing data (>10% of observations missing); and (iii) were collinear with other variables included in the model.
