# Supplementary material for: Integrated Source Case Investigation for Tuberculosis (TB) and HIV in the Caregivers and Household Contacts of Hospitalised Young Children Diagnosed with TB in South Africa: An Observational Study
Source: PLoS One. 2015 Sep 17;10(9):e0137518. doi: 10.1371/journal.pone.0137518 (PMC4574562; doi:10.1371/journal.pone.0137518)
Supplement: S4 Table — (DOCX) [file pone.0137518.s011.docx]

S4 Table. The effect of targeted screening on the yield of newly diagnosed TB in caregivers who report TB symptoms and/or are HIV infected [% (n)]

|  | **Detected cases of newly‑diagnosed TB**  **[% yield (# newly‑diagnosed TB cases / # caregivers)]** | **Missed cases of newly‑diagnosed TB**  **[% yield (# newly‑diagnosed TB cases / # caregivers)]** |
| --- | --- | --- |
| All caregivers | 4.0% (23/576) | - |
| HIV-infected caregivers | 4.9% (18/364) | 2.4% (5/212) |
| Caregivers reporting TB symptoms | 9.3% (13/140) | 2.3% (10/436) |
| HIV‑infected caregivers reporting TB symptoms | 12.1% (12/99) | 2.3% (11/477) |
